# Supplementary figures and images for: A novel model of glioblastoma recurrence to identify therapeutic vulnerabilities
Source: EMBO Mol Med. 2025 Apr 28;17(6):1325–54. doi: 10.1038/s44321-025-00237-z (PMC12162887; doi:10.1038/s44321-025-00237-z)

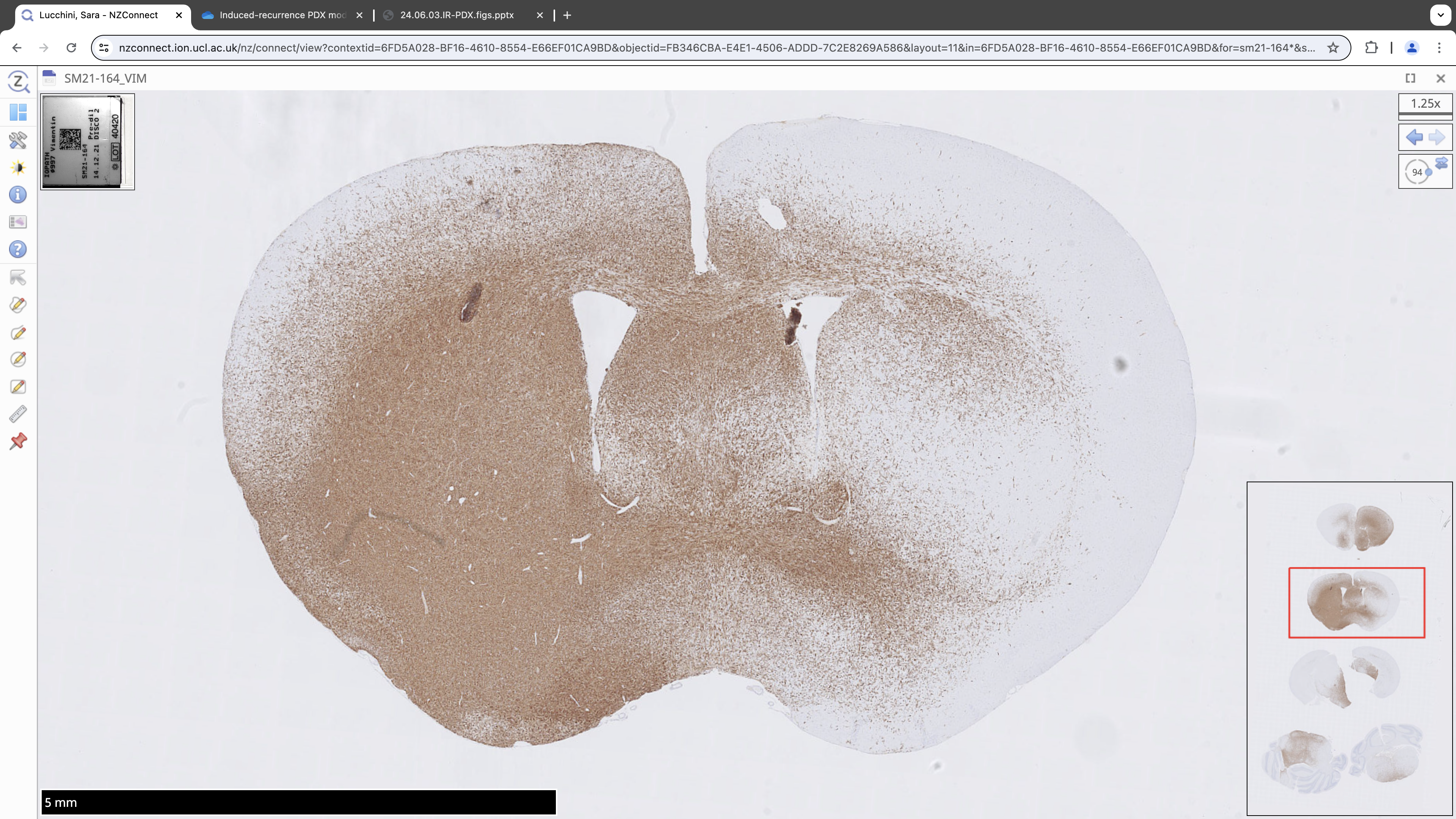

Supplement: Supplementary file 2 — Source data Fig. 1 [file 44321_2025_237_MOESM2_ESM.zip › Figure 1/1E/VIM_whole brain_XGBM67.png]

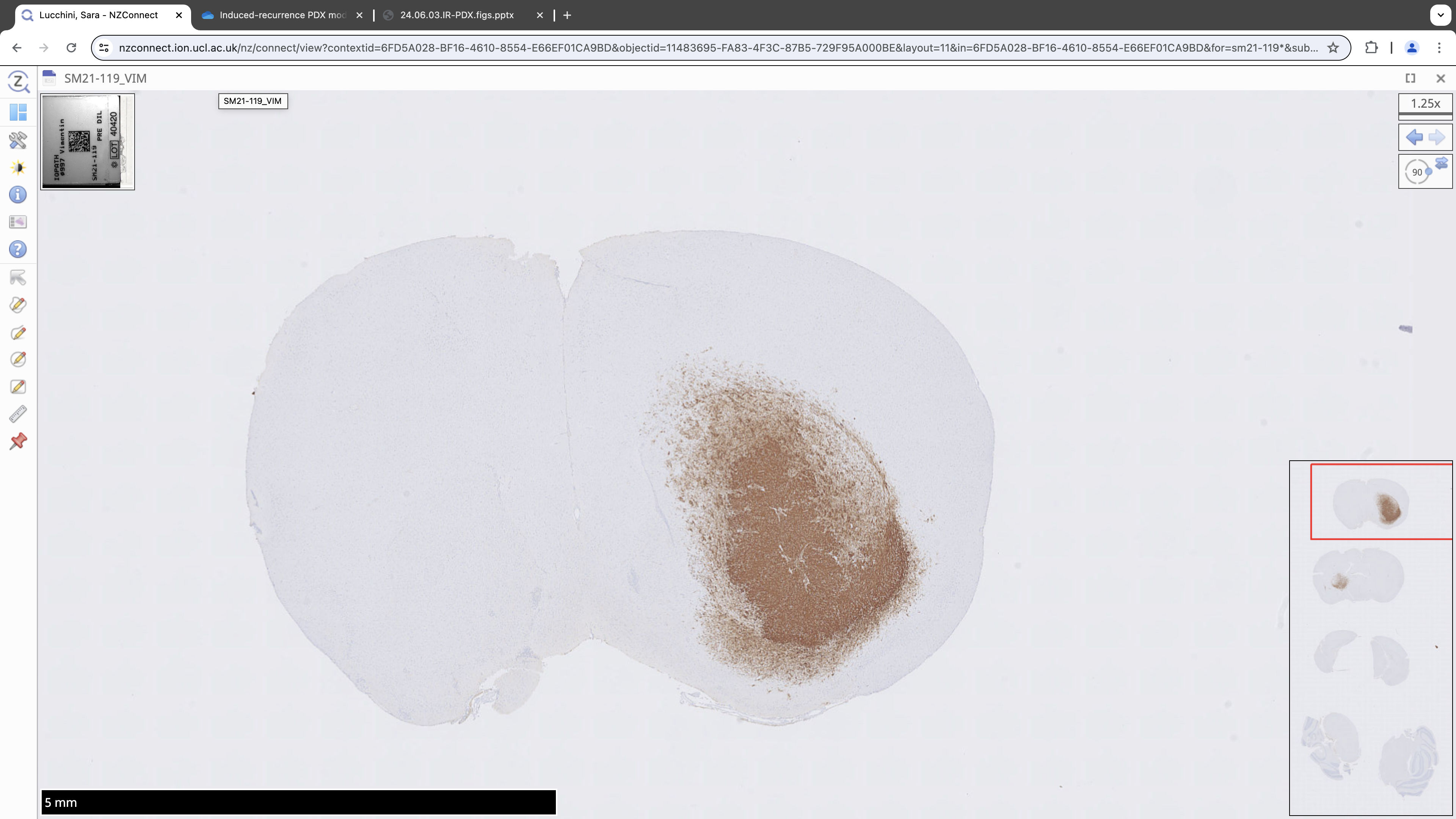

Supplement: Supplementary file 2 — Source data Fig. 1 [file 44321_2025_237_MOESM2_ESM.zip › Figure 1/1E/VIM_whole brain_XGBM39R.png]

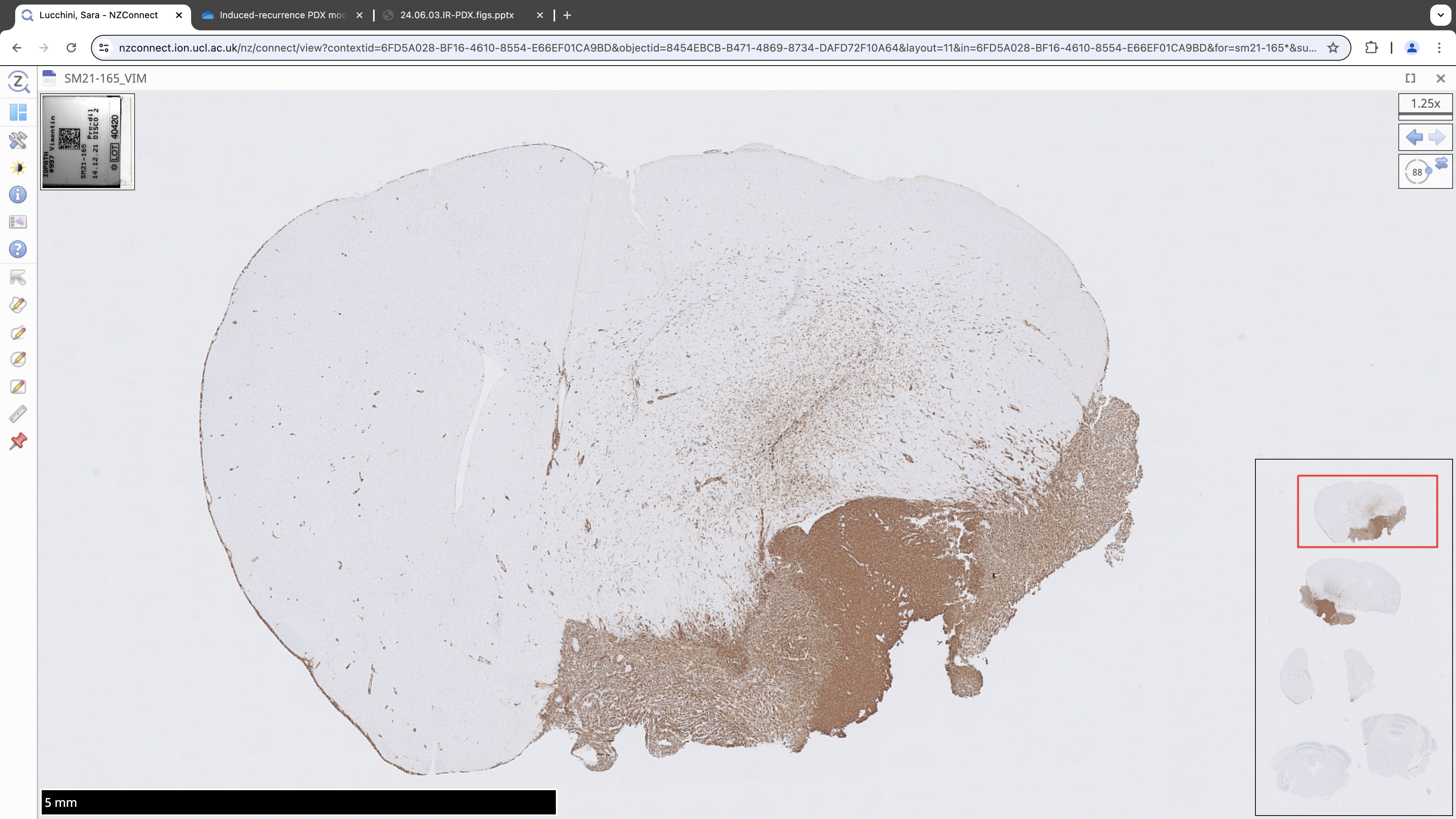

Supplement: Supplementary file 2 — Source data Fig. 1 [file 44321_2025_237_MOESM2_ESM.zip › Figure 1/1E/VIM_whole brain_XGBM39.png]

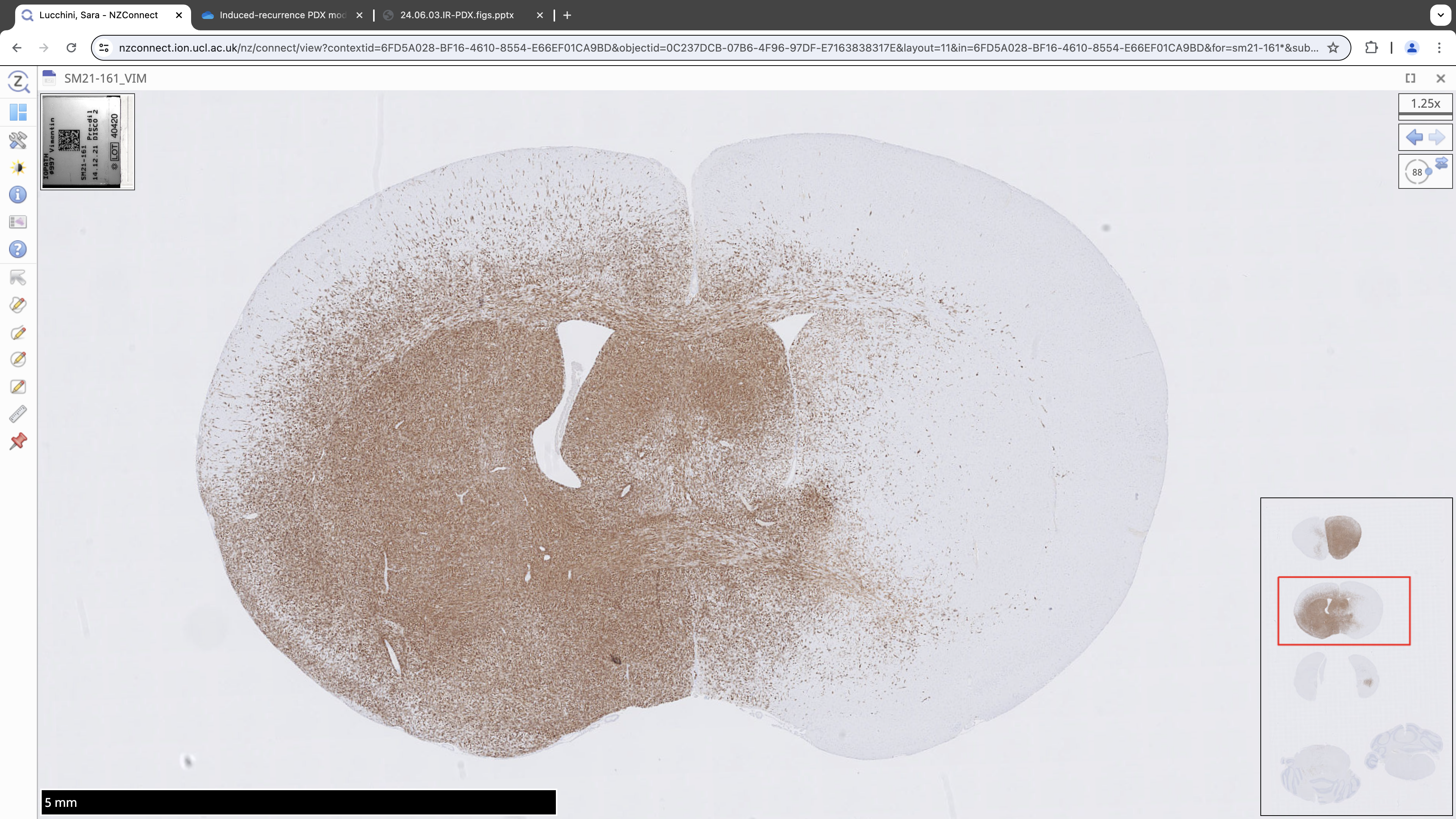

Supplement: Supplementary file 2 — Source data Fig. 1 [file 44321_2025_237_MOESM2_ESM.zip › Figure 1/1E/VIM_whole brain_XGBM67R.png]

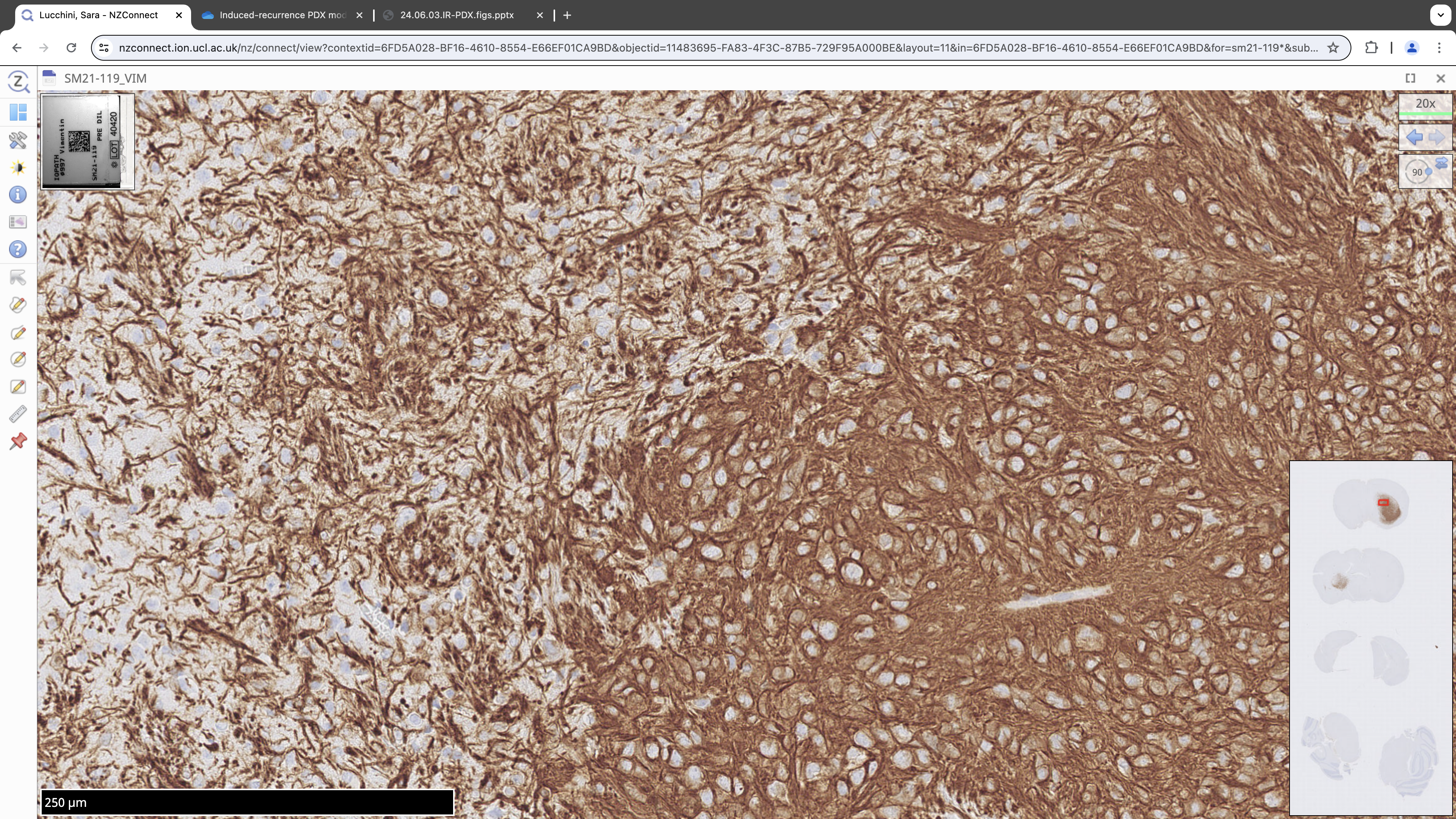

Supplement: Supplementary file 2 — Source data Fig. 1 [file 44321_2025_237_MOESM2_ESM.zip › Figure 1/1C/hVim_20X_XGBM39R.png]

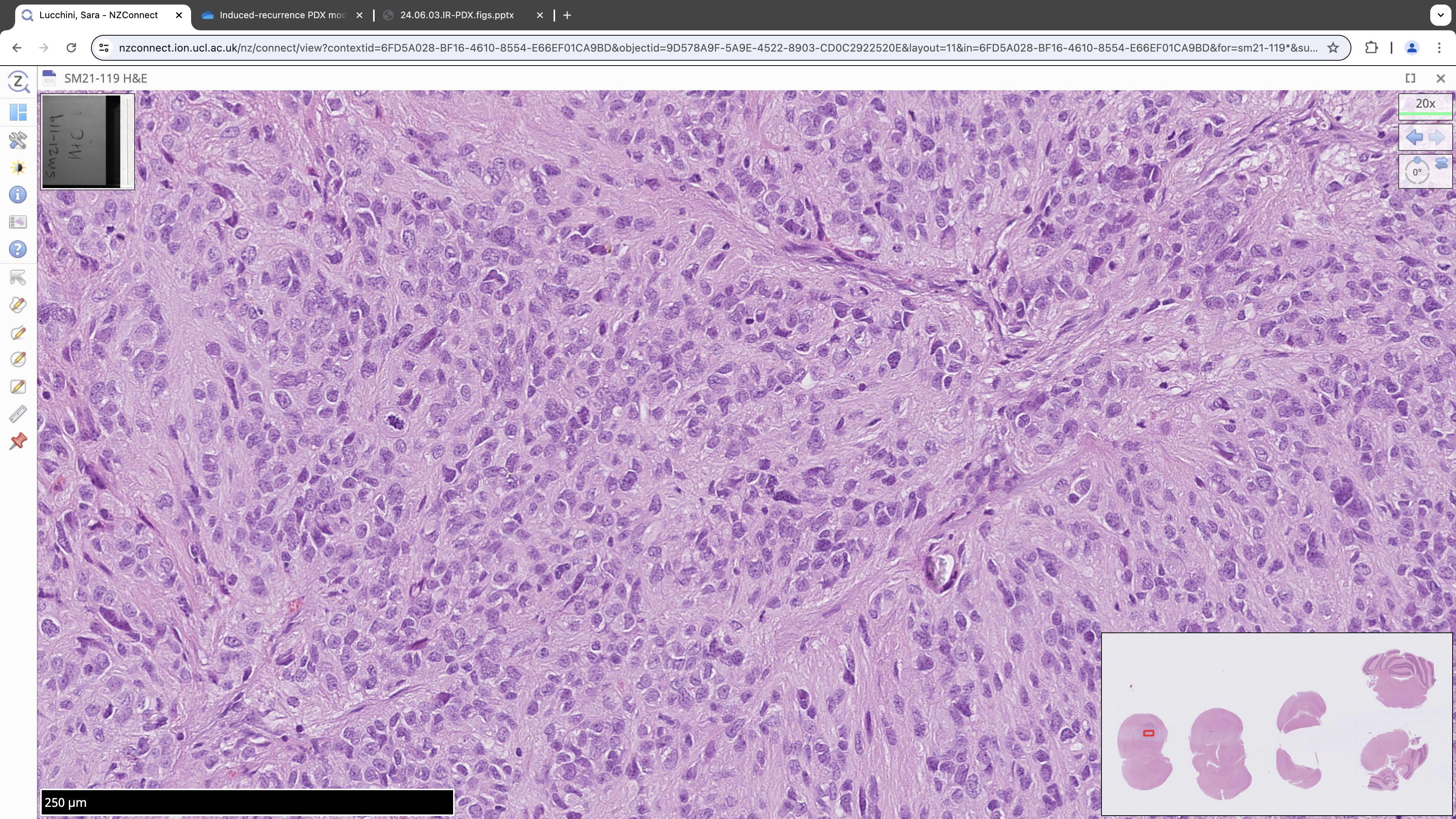

Supplement: Supplementary file 2 — Source data Fig. 1 [file 44321_2025_237_MOESM2_ESM.zip › Figure 1/1C/H&E_20X_XGBM39R.png]

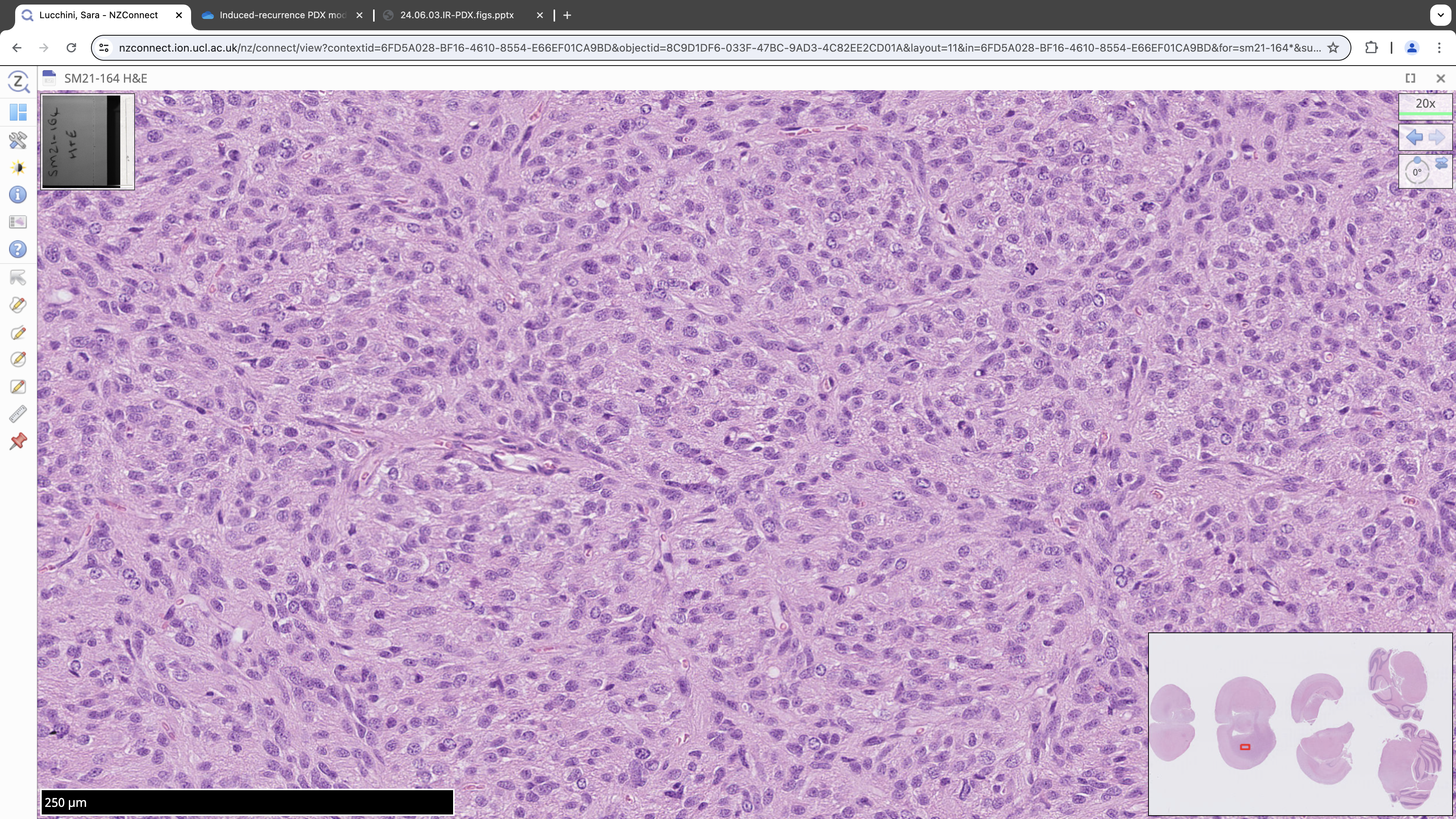

Supplement: Supplementary file 2 — Source data Fig. 1 [file 44321_2025_237_MOESM2_ESM.zip › Figure 1/1C/H&E_20X_XGBM67.png]

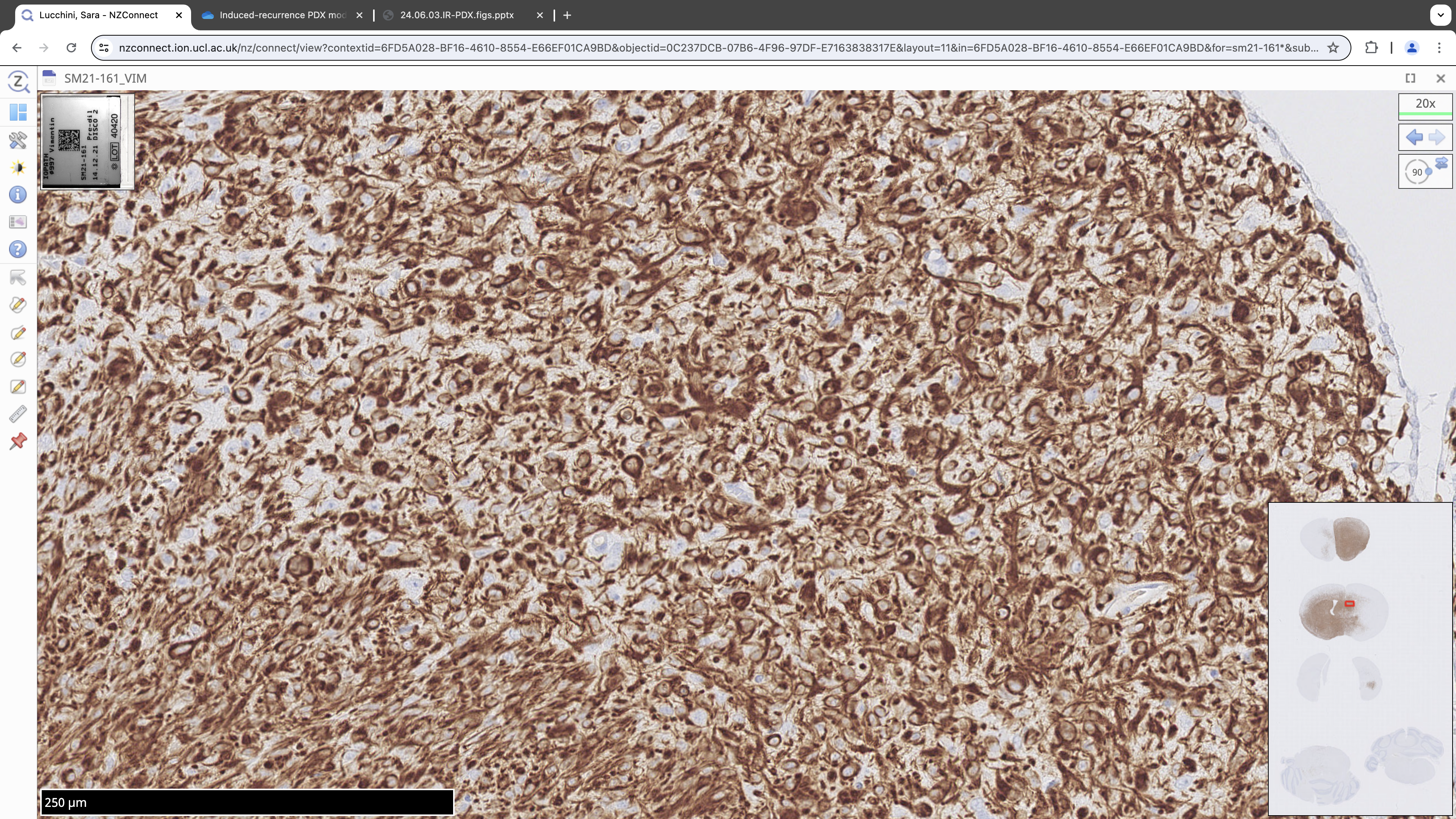

Supplement: Supplementary file 2 — Source data Fig. 1 [file 44321_2025_237_MOESM2_ESM.zip › Figure 1/1C/hVim_20X_XGBM67R.png]

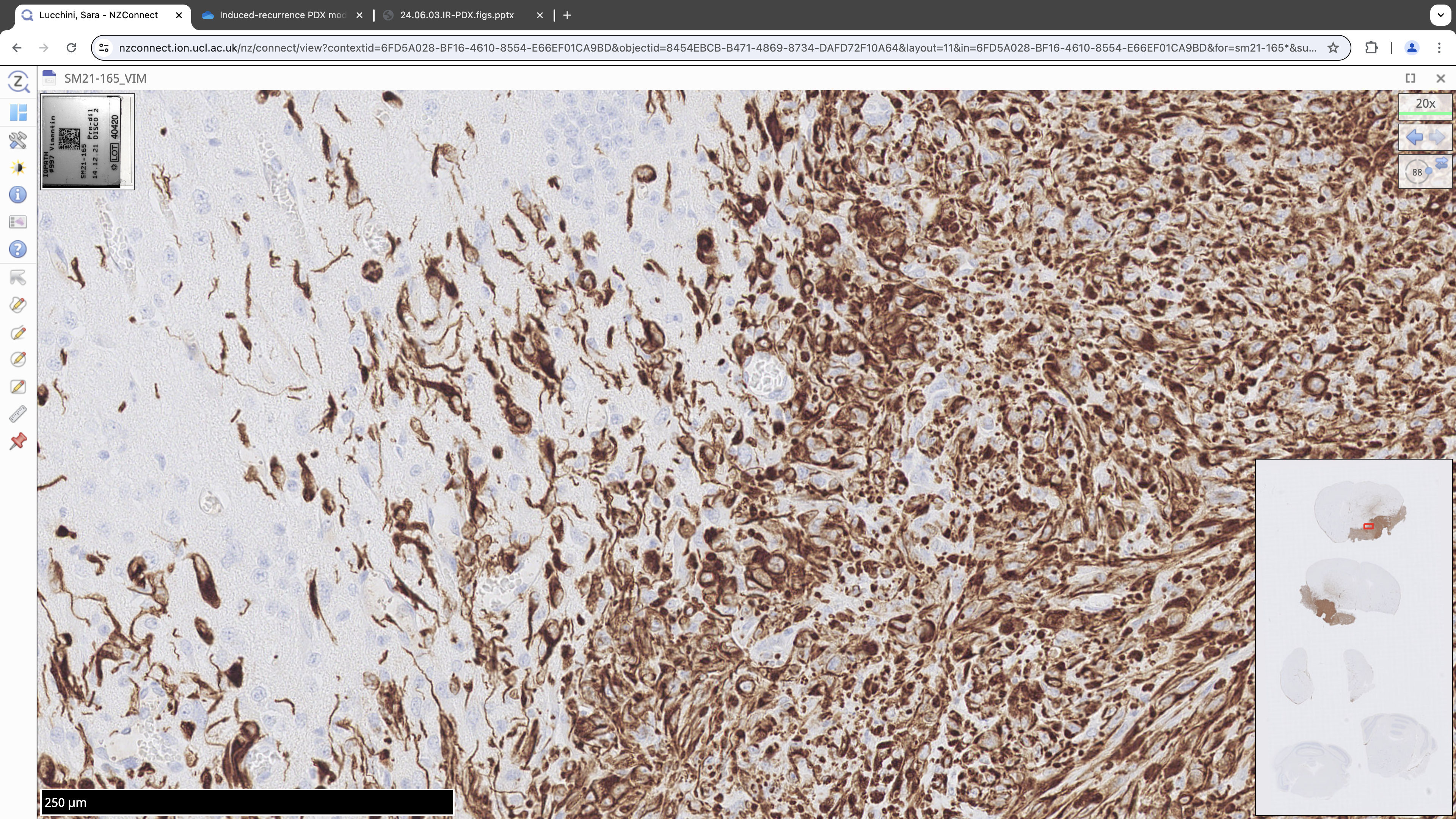

Supplement: Supplementary file 2 — Source data Fig. 1 [file 44321_2025_237_MOESM2_ESM.zip › Figure 1/1C/hVim_20X_XGBM39.png]

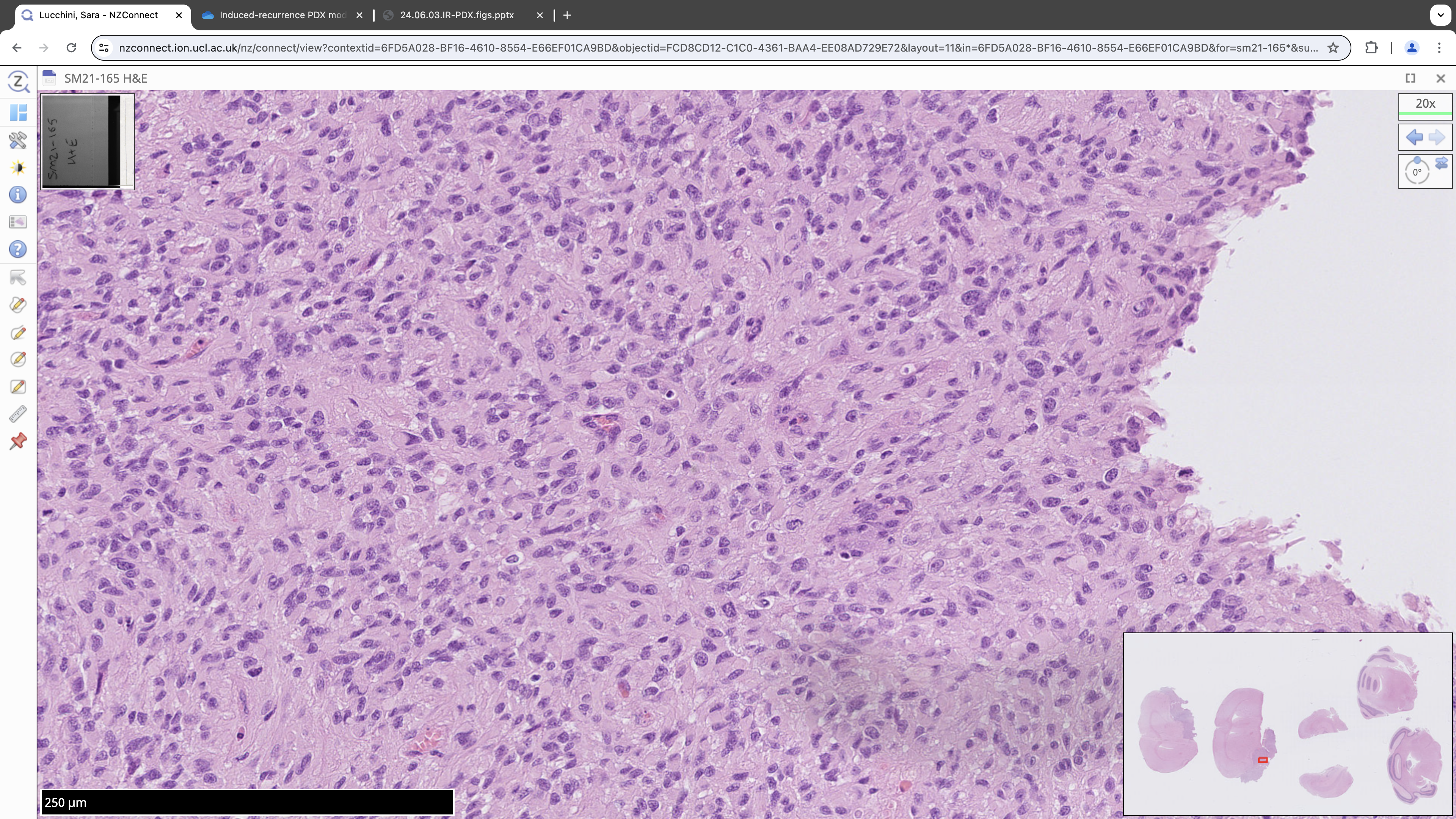

Supplement: Supplementary file 2 — Source data Fig. 1 [file 44321_2025_237_MOESM2_ESM.zip › Figure 1/1C/H&E_20X_XGBM39.png]

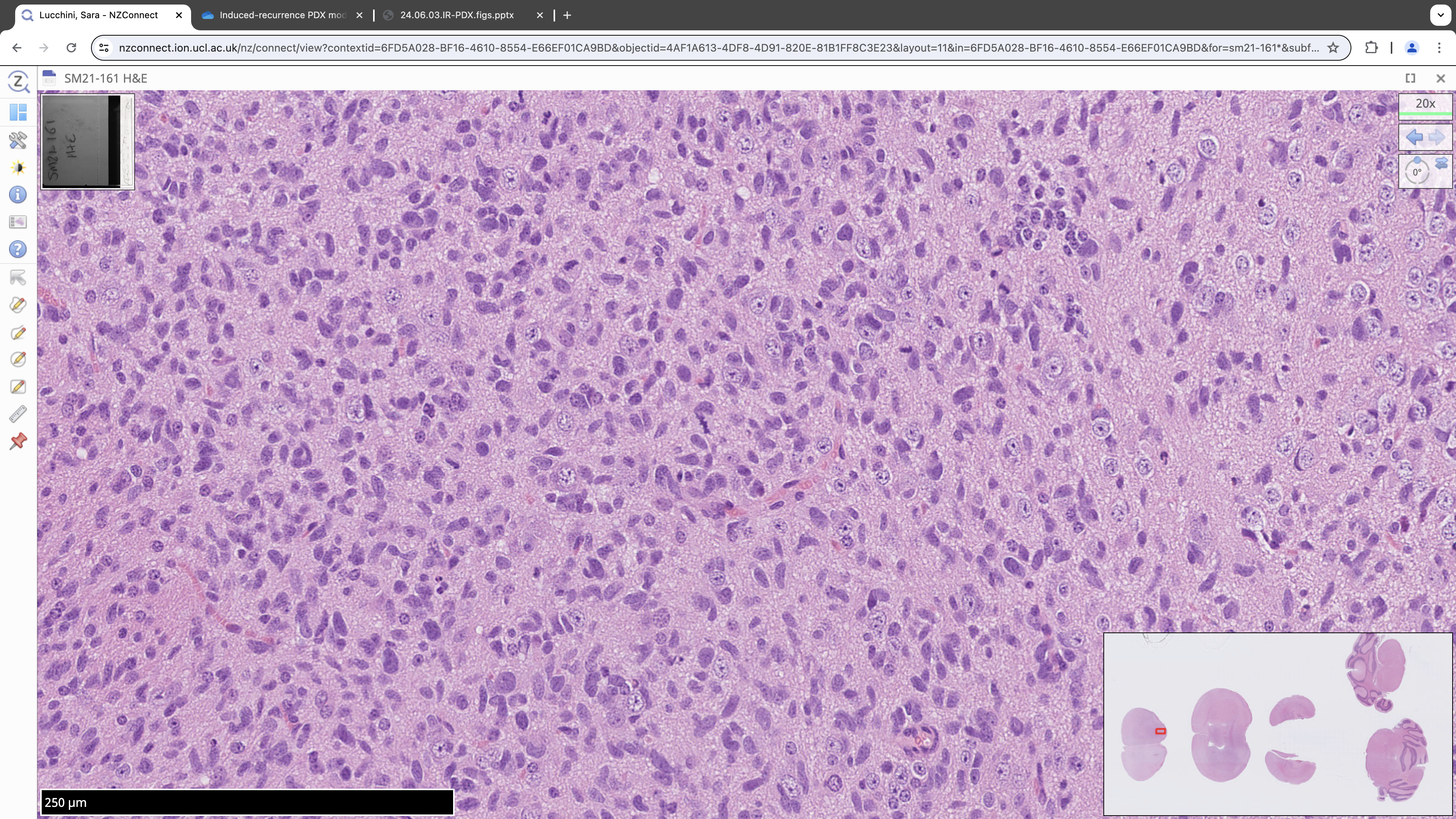

Supplement: Supplementary file 2 — Source data Fig. 1 [file 44321_2025_237_MOESM2_ESM.zip › Figure 1/1C/H&E_20X_XGBM67R.png]

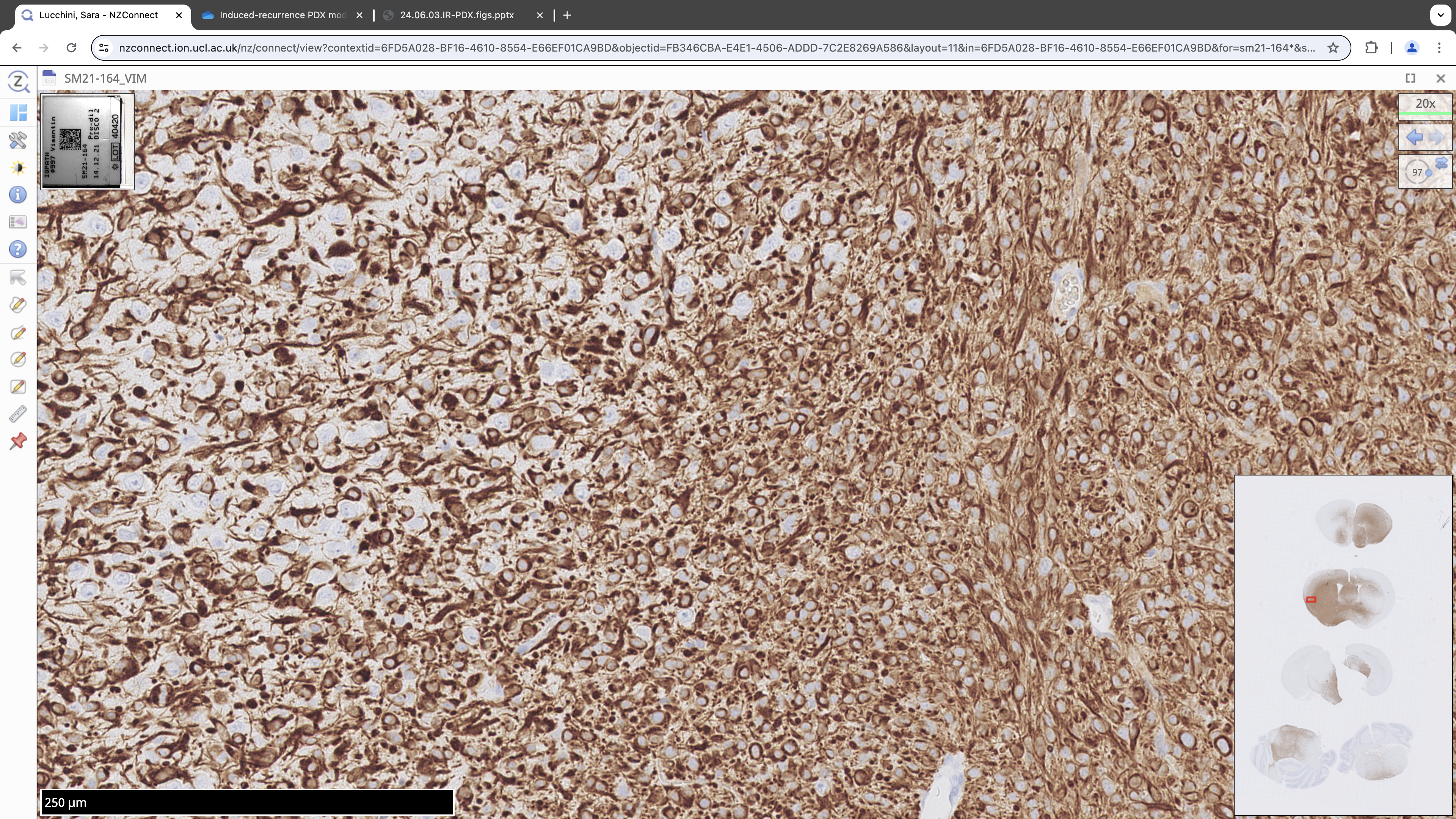

Supplement: Supplementary file 2 — Source data Fig. 1 [file 44321_2025_237_MOESM2_ESM.zip › Figure 1/1C/hVim_20X_XGBM67.png]

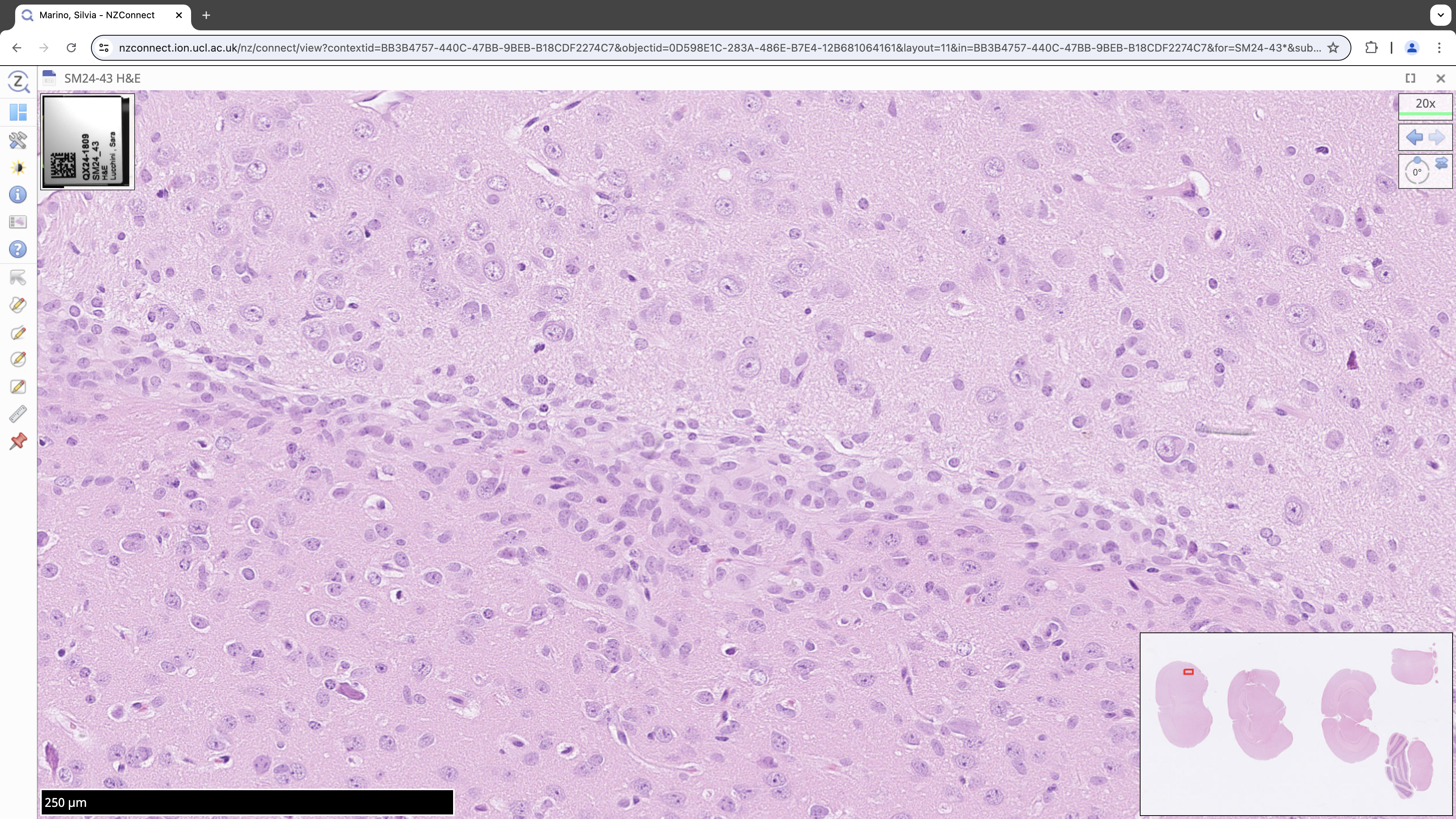

Supplement: Supplementary file 3 — Source data Fig. 2 [file 44321_2025_237_MOESM3_ESM.zip › Figure 2/2D/H&E_20X_IR-GBM39.png]

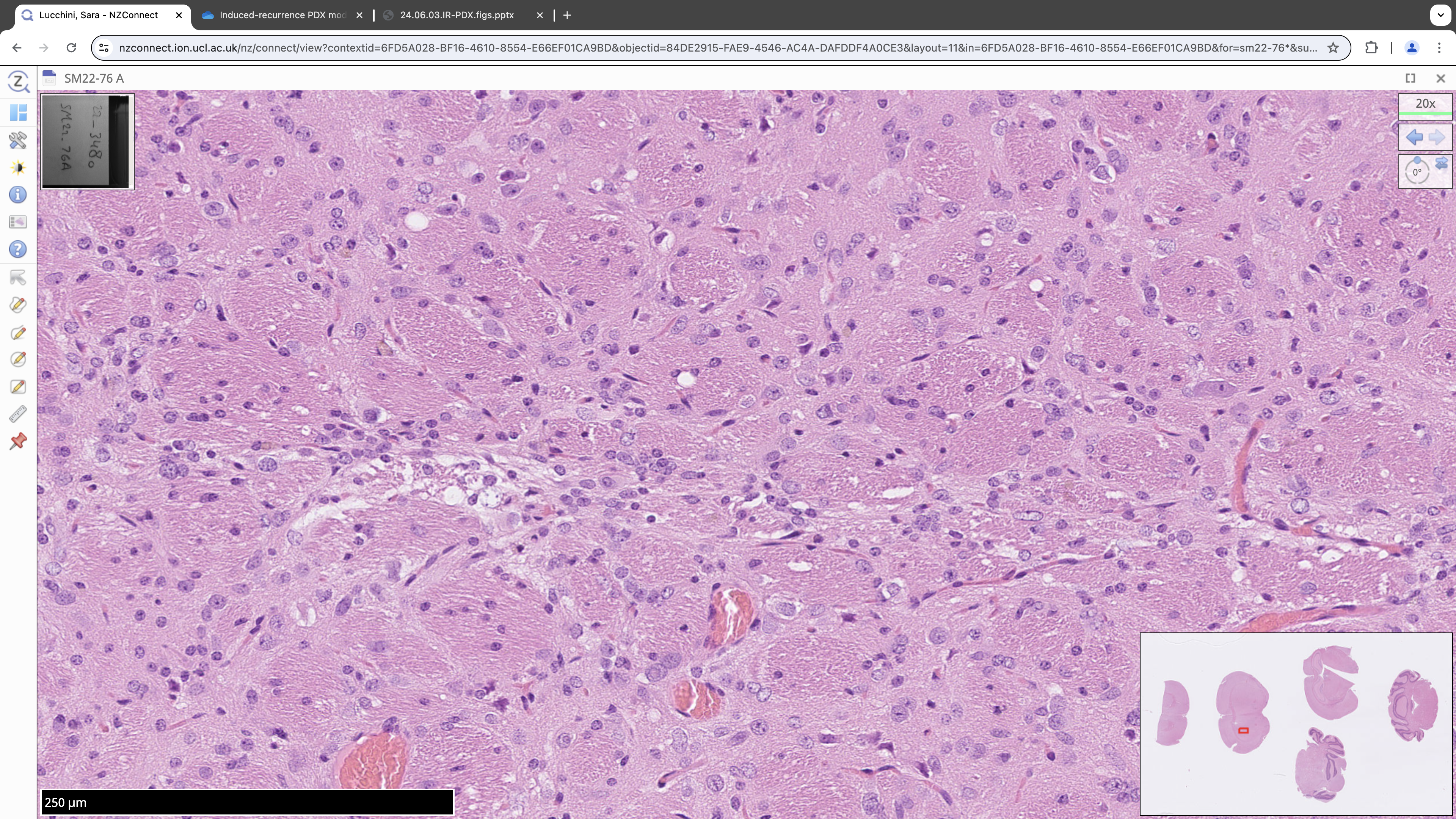

Supplement: Supplementary file 3 — Source data Fig. 2 [file 44321_2025_237_MOESM3_ESM.zip › Figure 2/2D/H&E_20X_IR-GBM67.png]

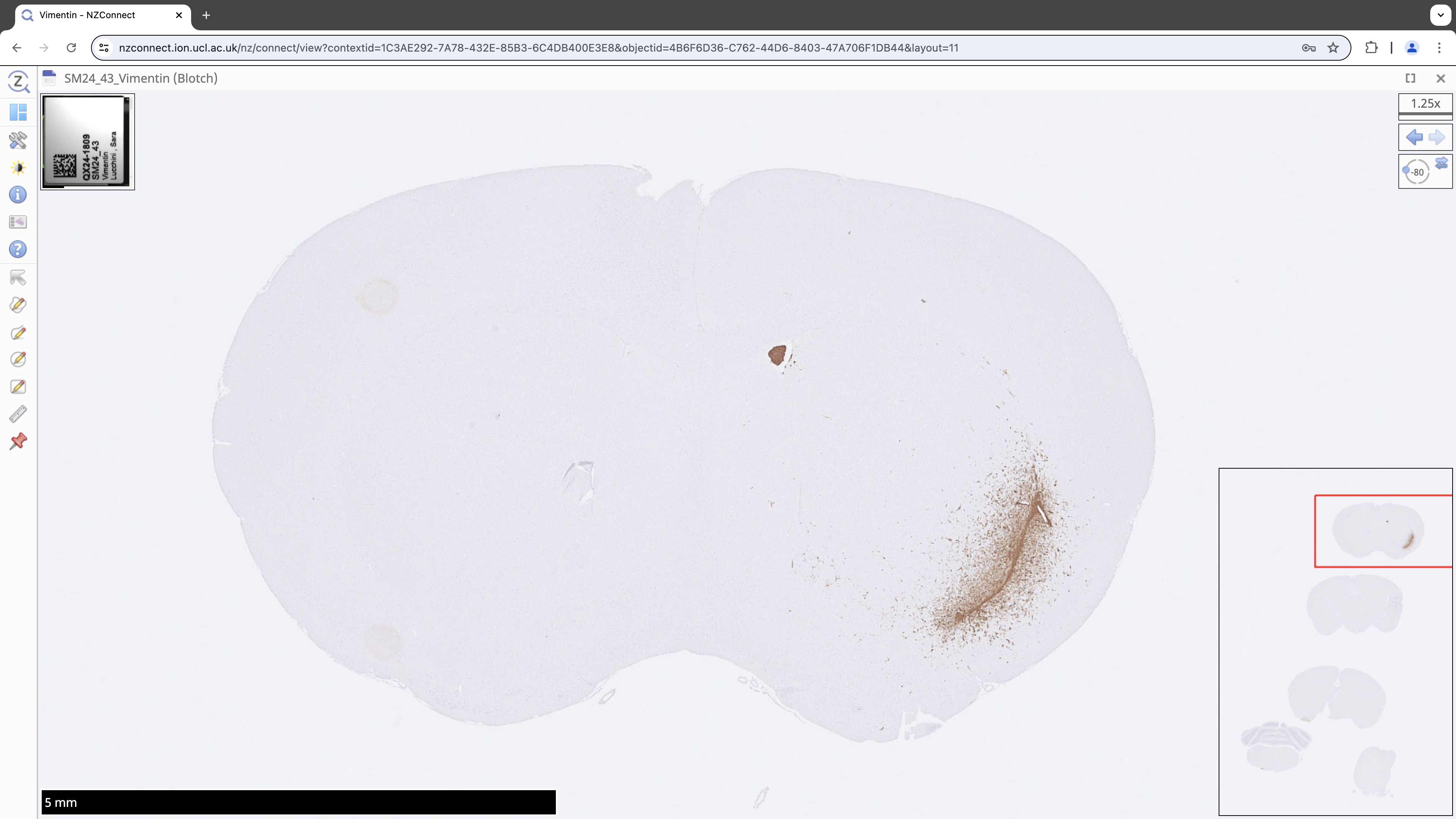

Supplement: Supplementary file 3 — Source data Fig. 2 [file 44321_2025_237_MOESM3_ESM.zip › Figure 2/2D/VIM_whole brain_IR-GBM39.png]

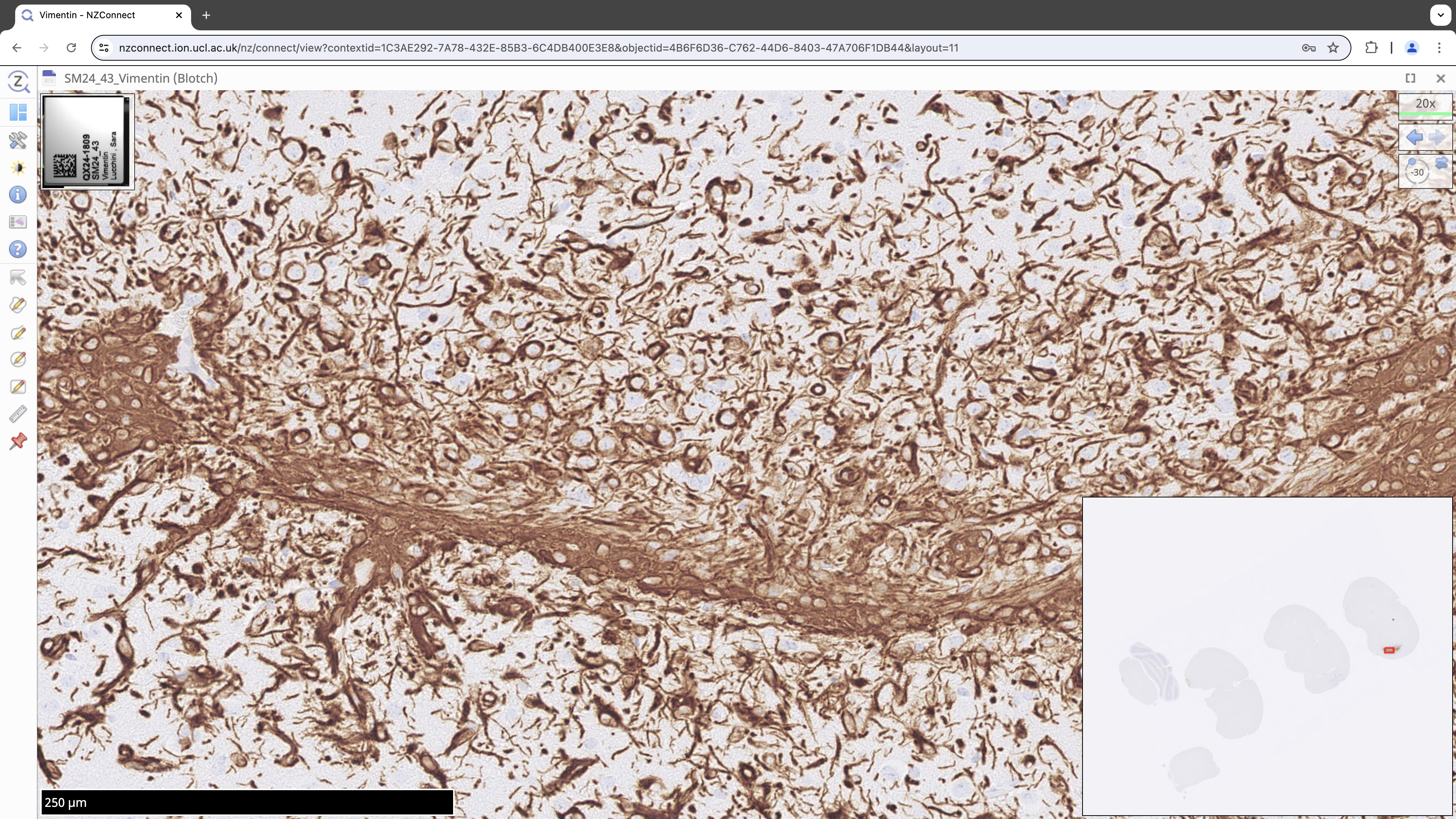

Supplement: Supplementary file 3 — Source data Fig. 2 [file 44321_2025_237_MOESM3_ESM.zip › Figure 2/2D/VIM_20X_IR-GBM39.png]

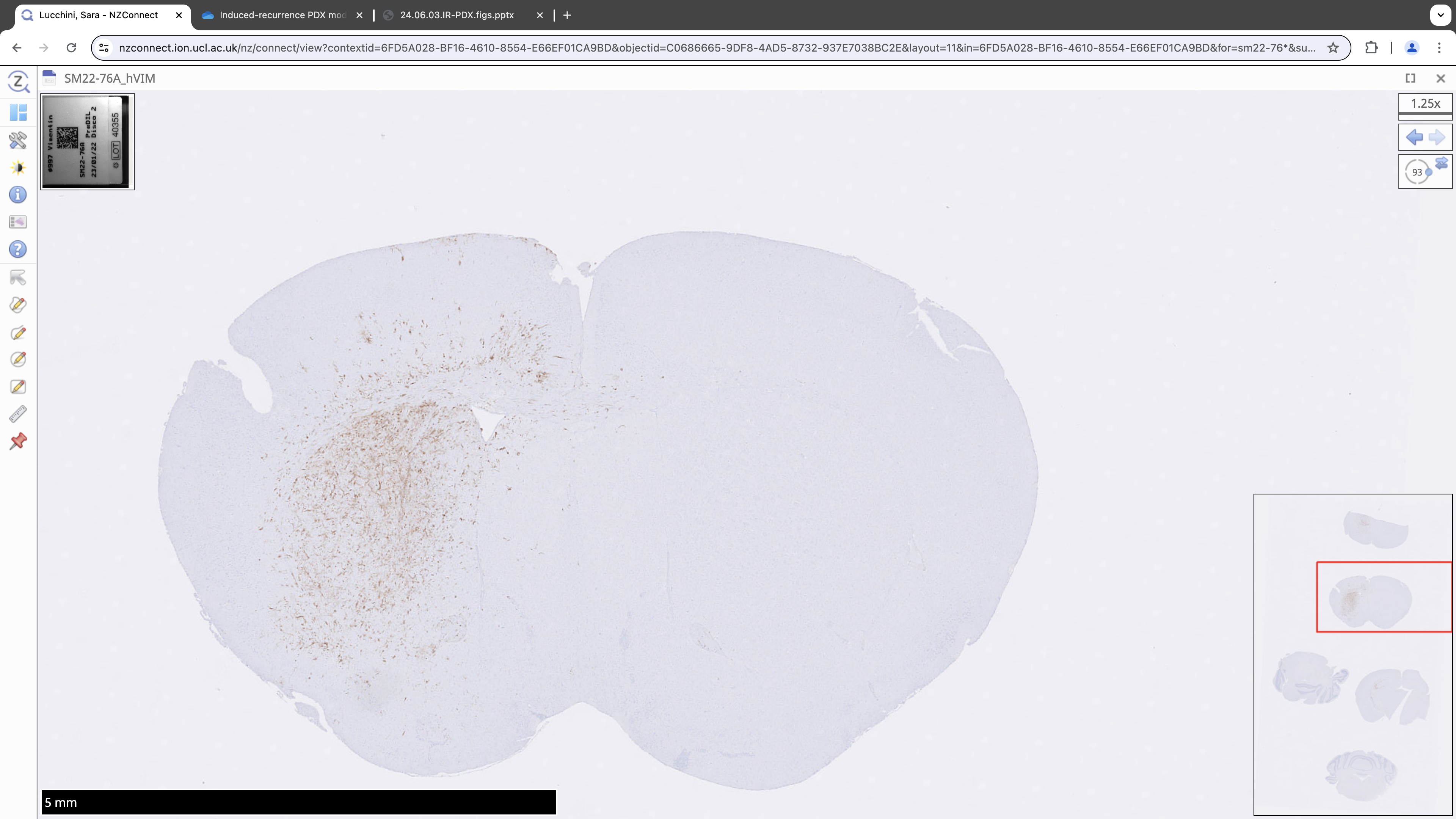

Supplement: Supplementary file 3 — Source data Fig. 2 [file 44321_2025_237_MOESM3_ESM.zip › Figure 2/2D/VIM_whole brain_IR-GBM67.png]

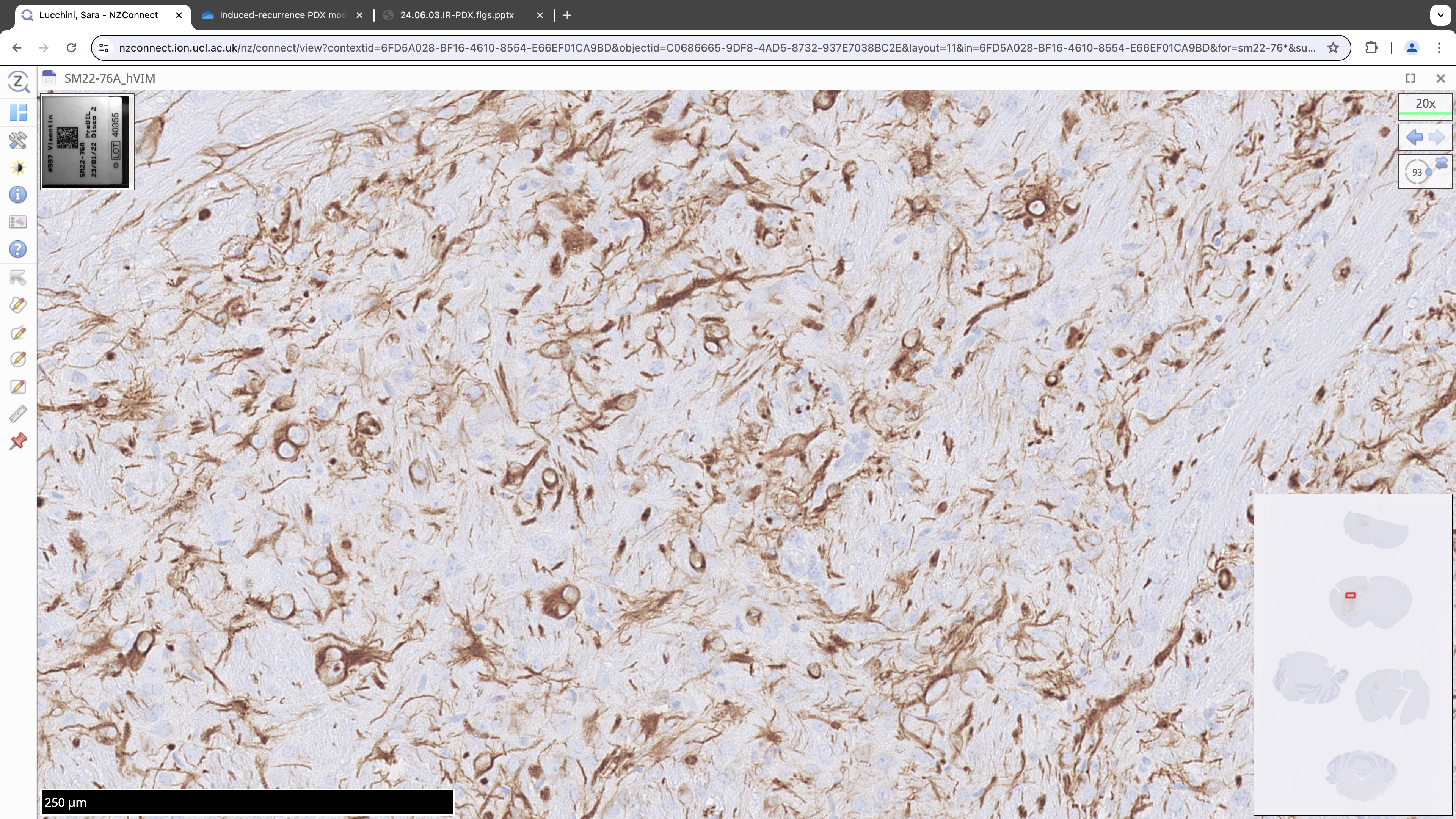

Supplement: Supplementary file 3 — Source data Fig. 2 [file 44321_2025_237_MOESM3_ESM.zip › Figure 2/2D/VIM_20X_IR-GBM67.png]

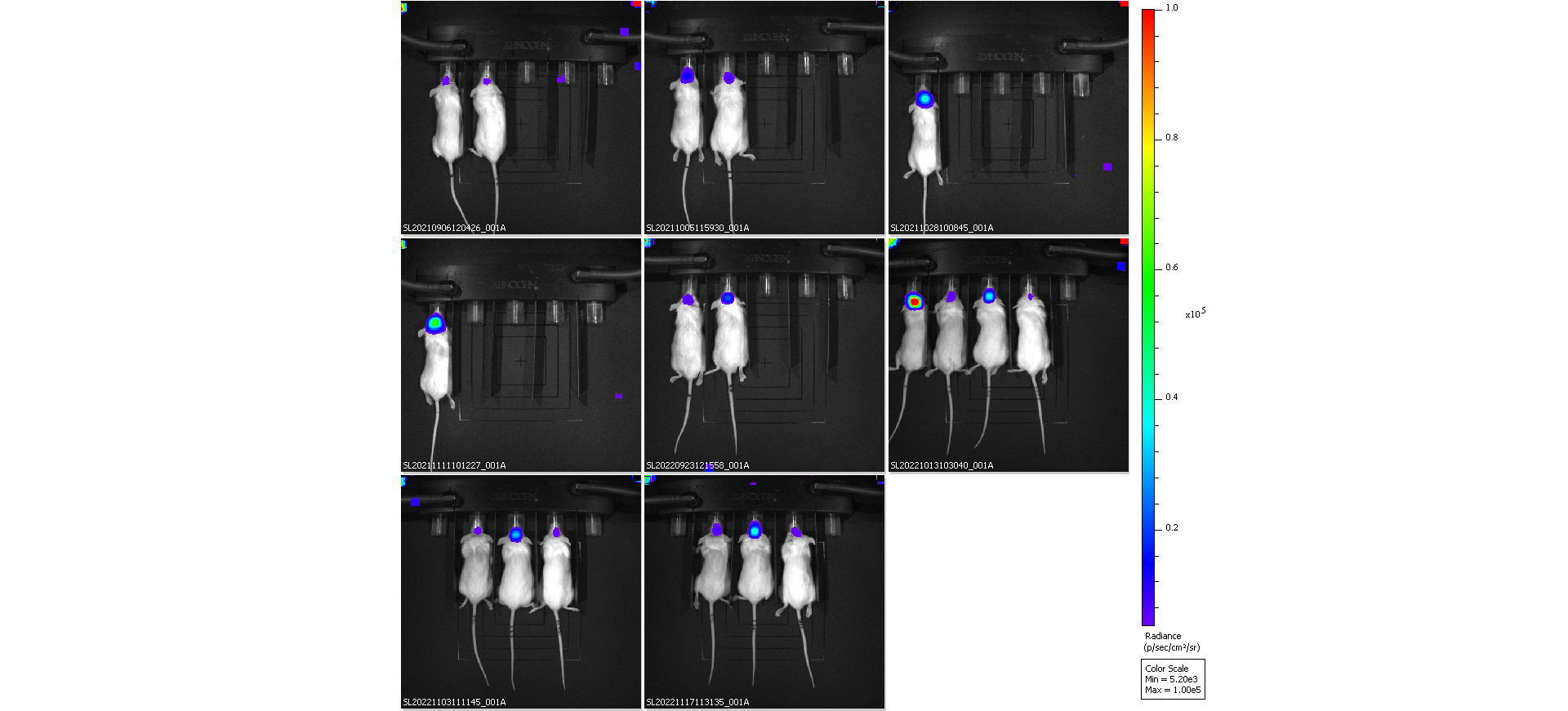

Supplement: Supplementary file 3 — Source data Fig. 2 [file 44321_2025_237_MOESM3_ESM.zip › Figure 2/2E/XGBM67 vs. IR-GBM67_same timepoints.tif]

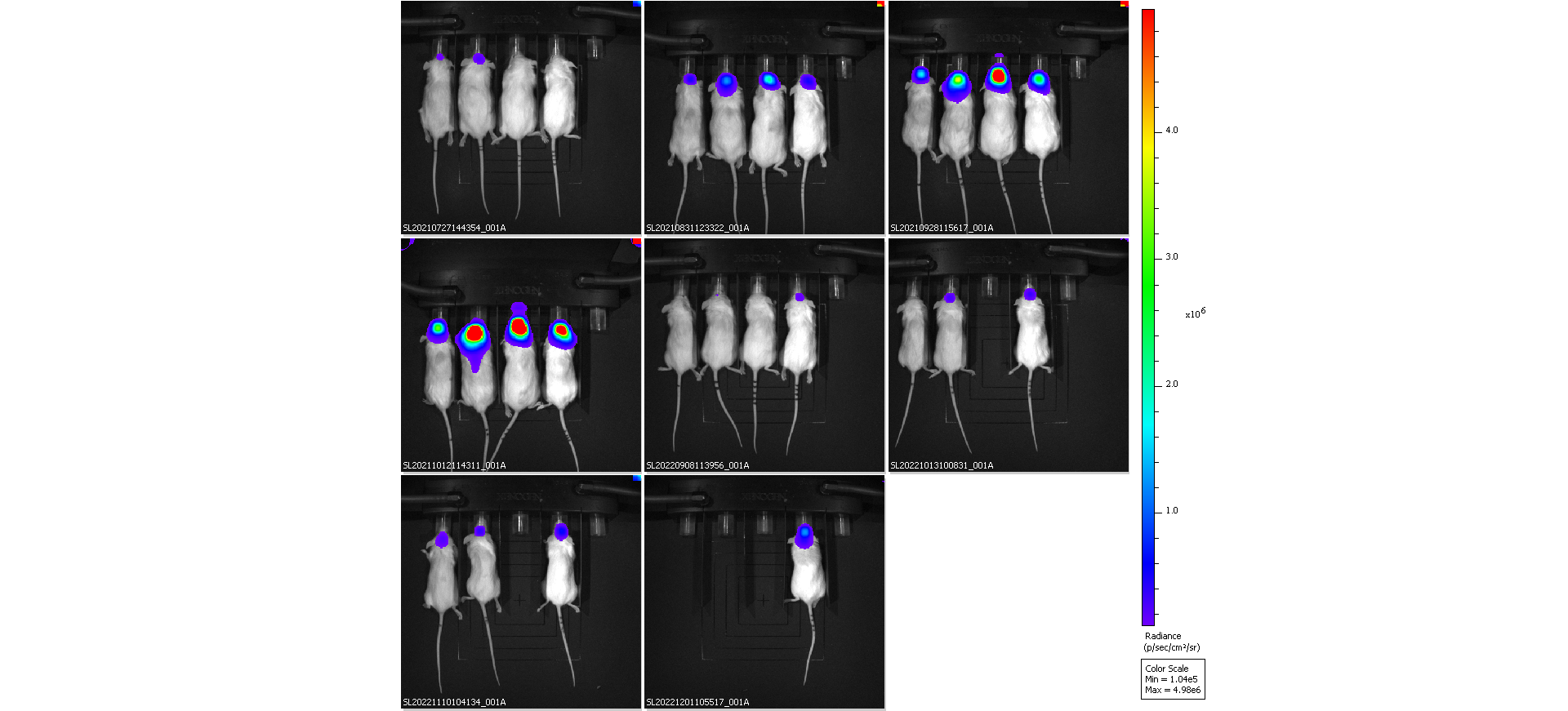

Supplement: Supplementary file 3 — Source data Fig. 2 [file 44321_2025_237_MOESM3_ESM.zip › Figure 2/2E/XGBM80 vs. IR-GBM80_same timepoints.tif]

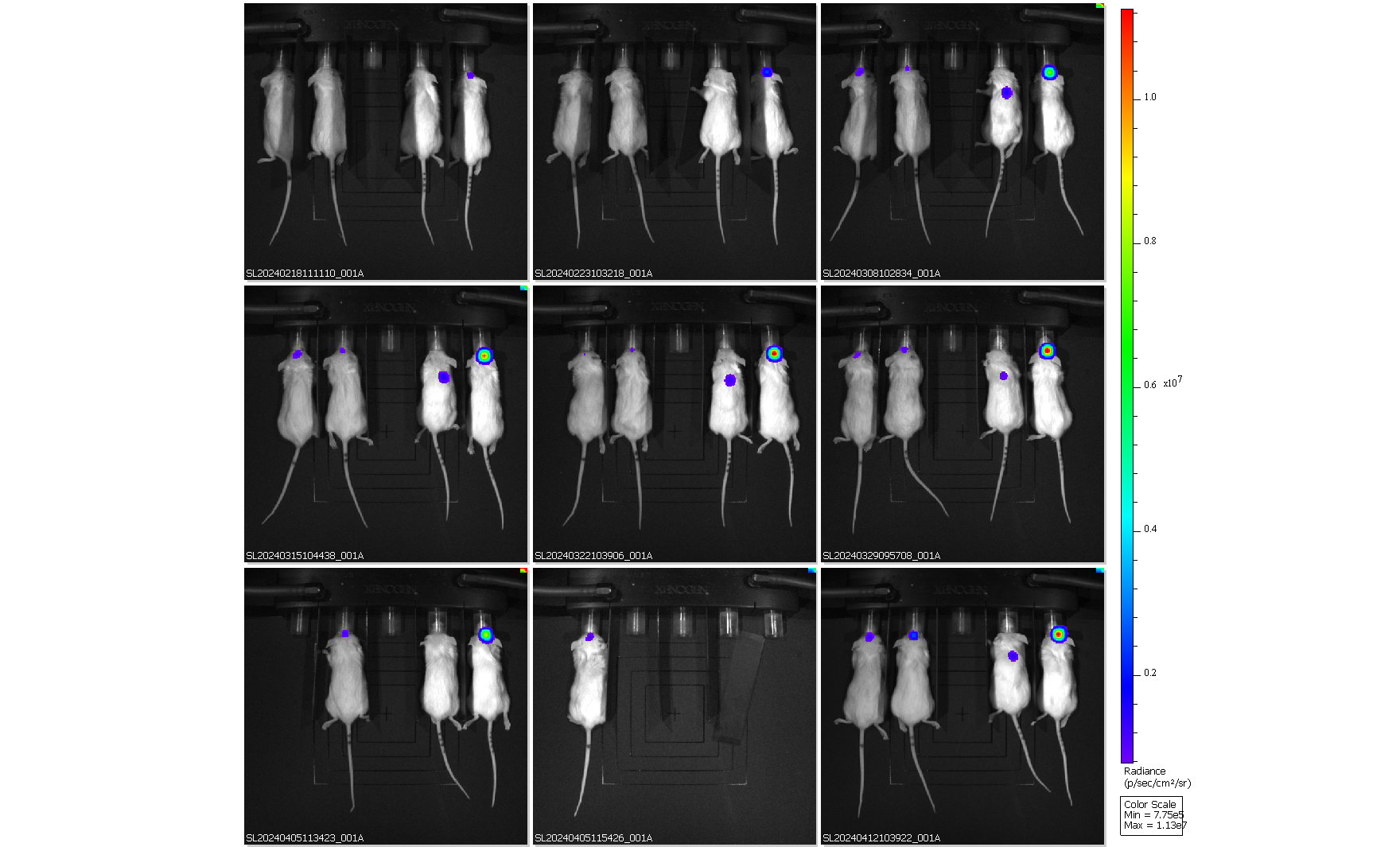

Supplement: Supplementary file 3 — Source data Fig. 2 [file 44321_2025_237_MOESM3_ESM.zip › Figure 2/2B/IR-GBM39_all time points.tif]

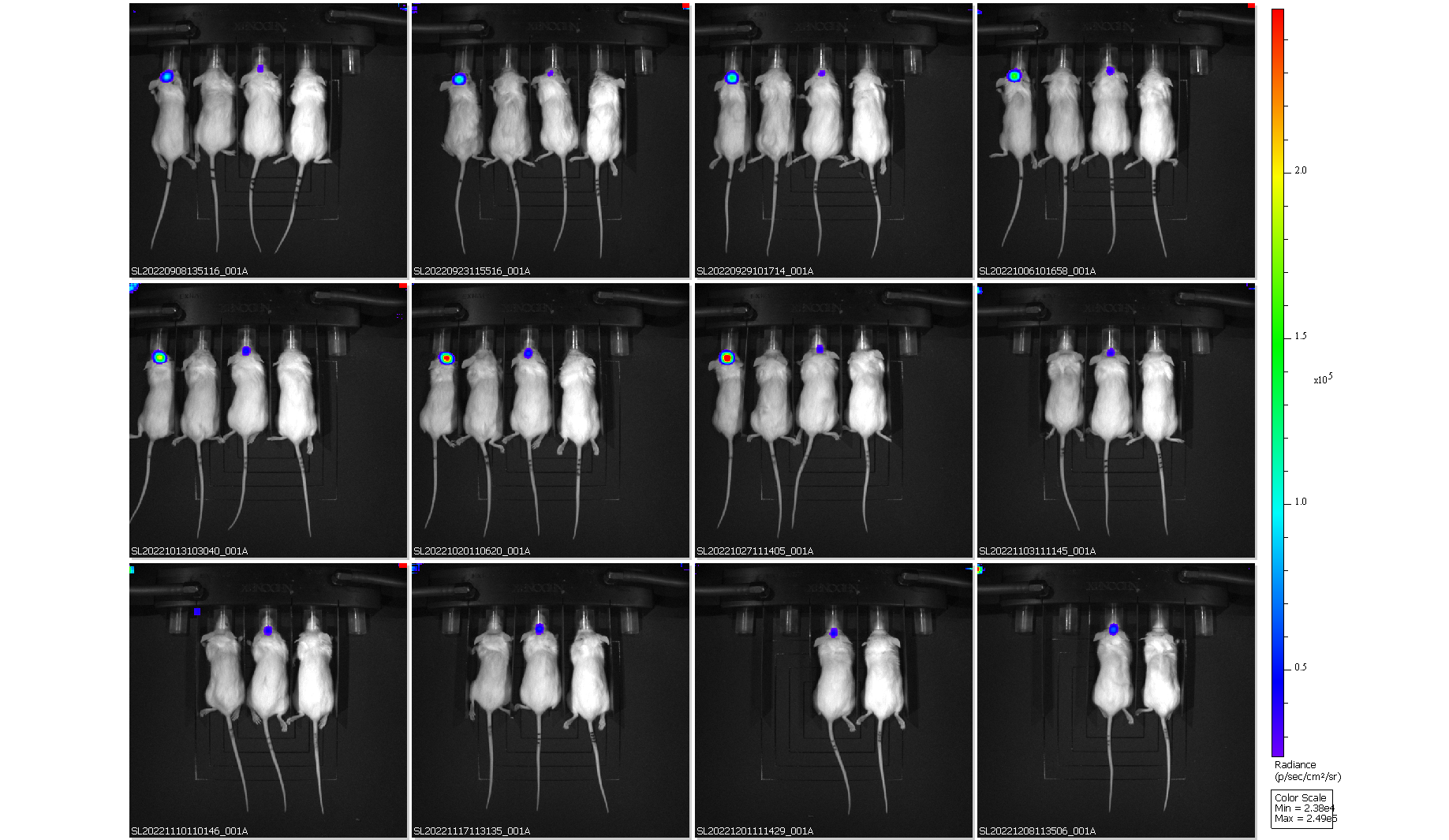

Supplement: Supplementary file 3 — Source data Fig. 2 [file 44321_2025_237_MOESM3_ESM.zip › Figure 2/2B/IR-GBM67_all timepoints.tif]

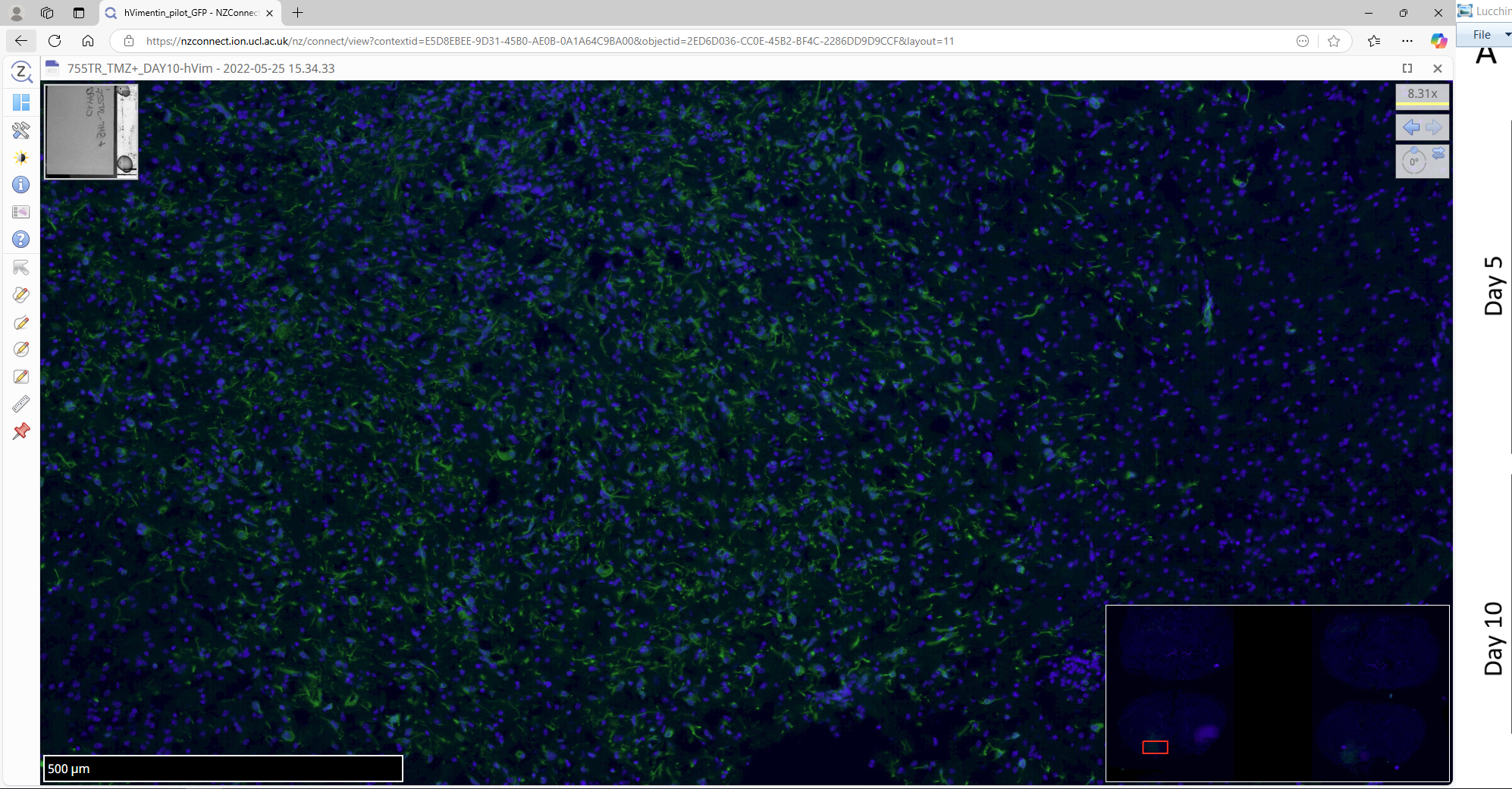

Supplement: Supplementary file 5 — EV Figure Source Data [file 44321_2025_237_MOESM5_ESM.zip › Figure EV2/EV2A/hVim_fromNZC/Day10_TMZ25mg.Kg.PNG]

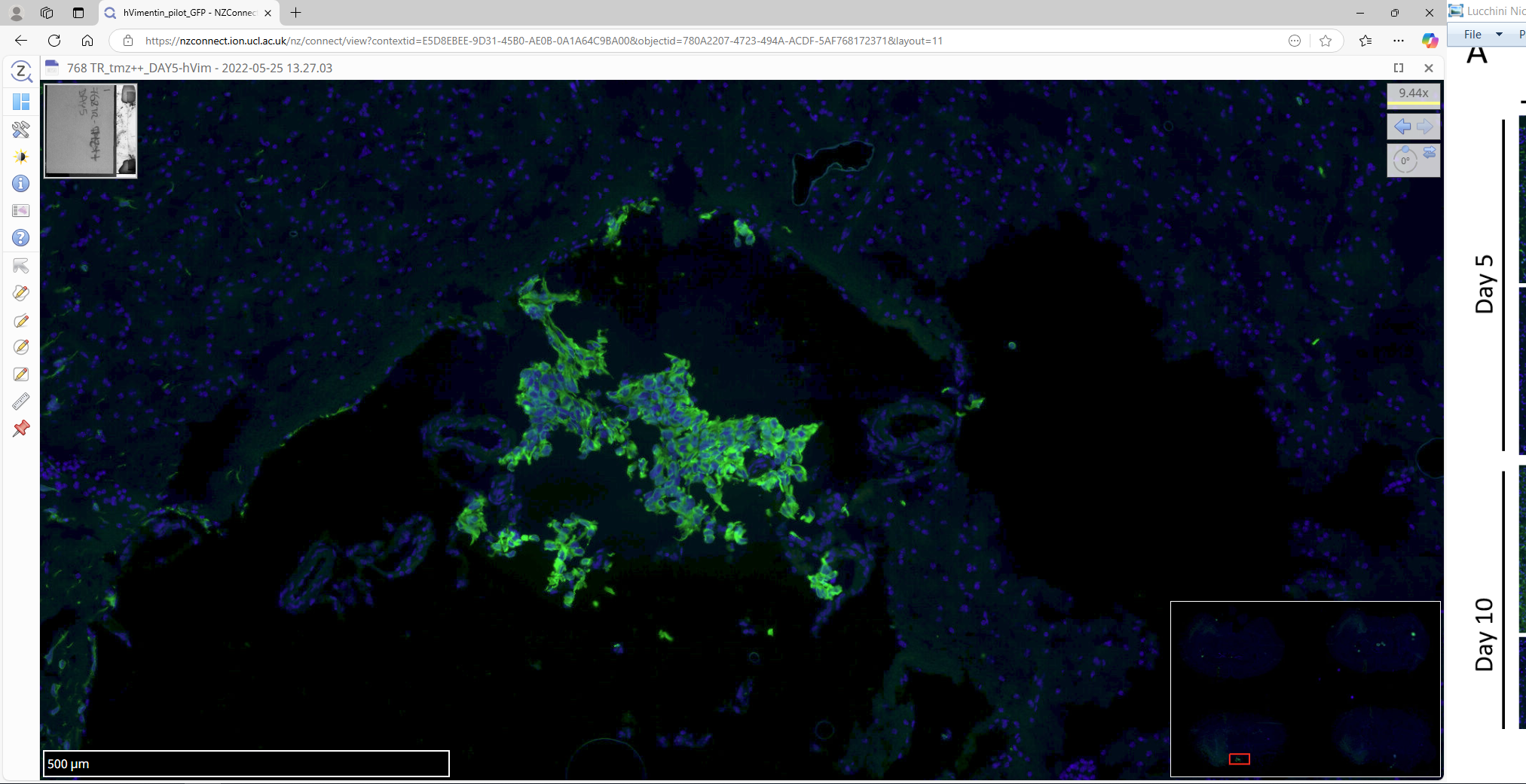

Supplement: Supplementary file 5 — EV Figure Source Data [file 44321_2025_237_MOESM5_ESM.zip › Figure EV2/EV2A/hVim_fromNZC/Day5_TMZ50mg.Kg.PNG]

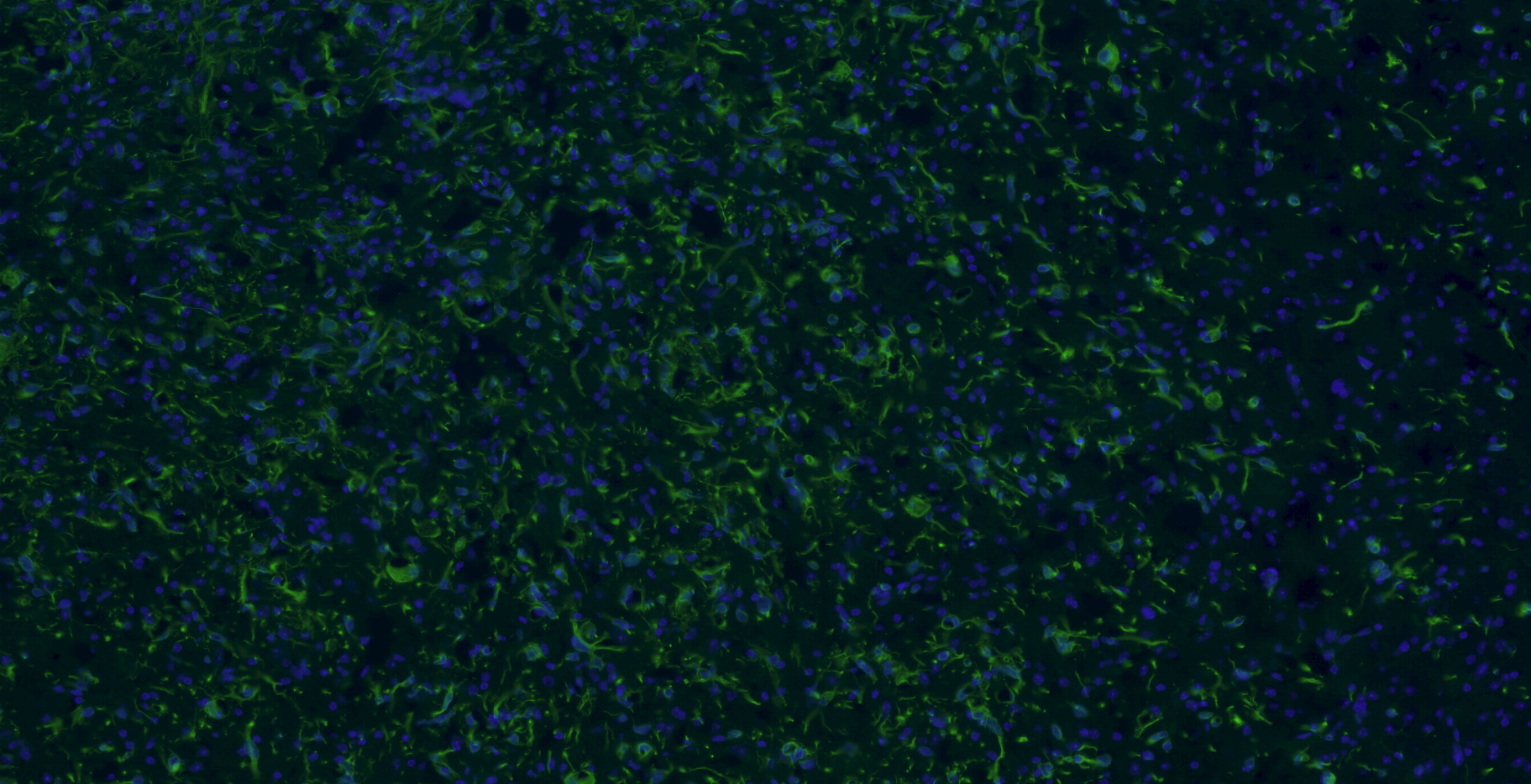

Supplement: Supplementary file 5 — EV Figure Source Data [file 44321_2025_237_MOESM5_ESM.zip › Figure EV2/EV2A/hVim_fromNZC/755TR_TMZ25_DAY10-hVim - 2022-05-25 15.34.33.jpg]

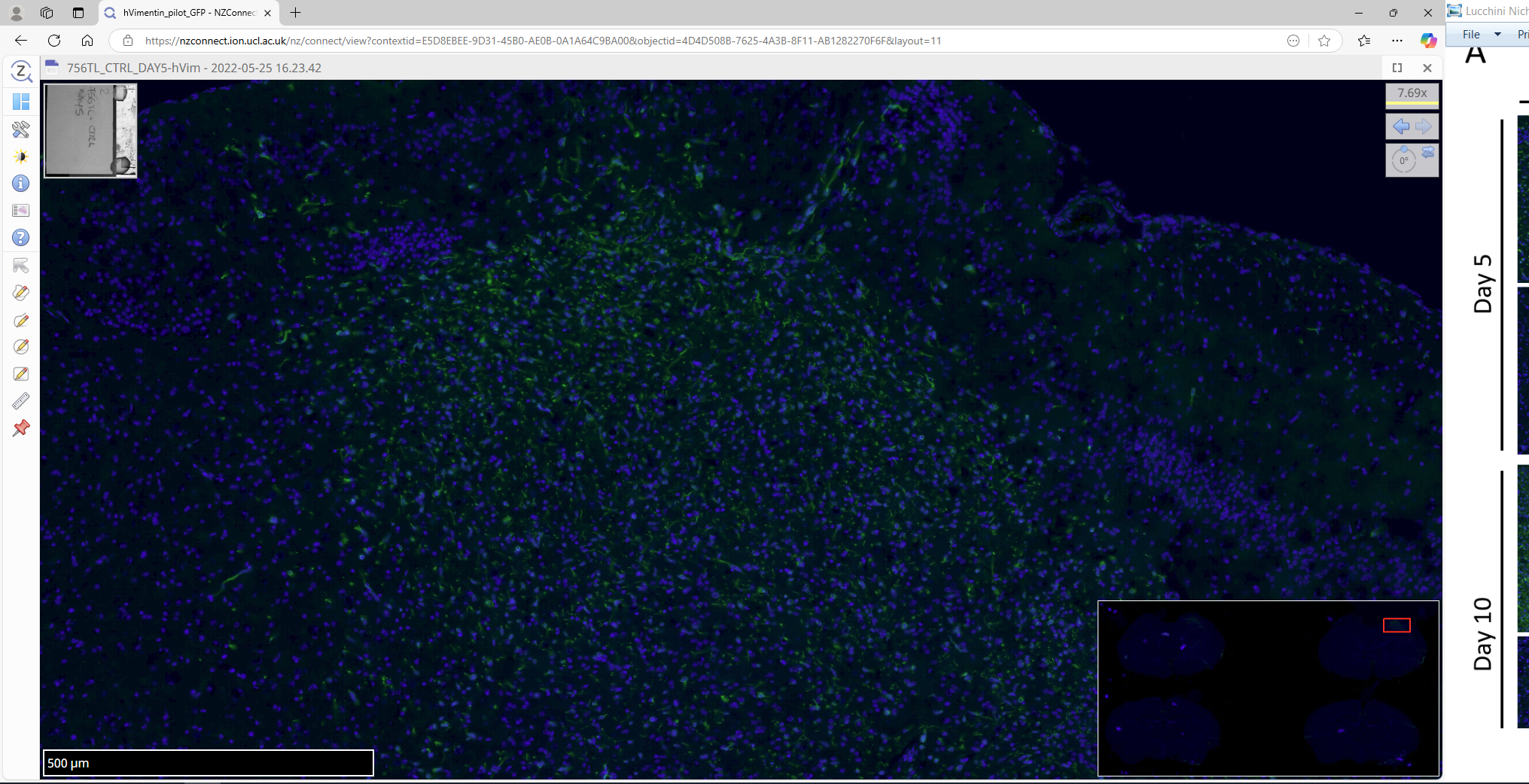

Supplement: Supplementary file 5 — EV Figure Source Data [file 44321_2025_237_MOESM5_ESM.zip › Figure EV2/EV2A/hVim_fromNZC/Day5_CTRL.PNG]

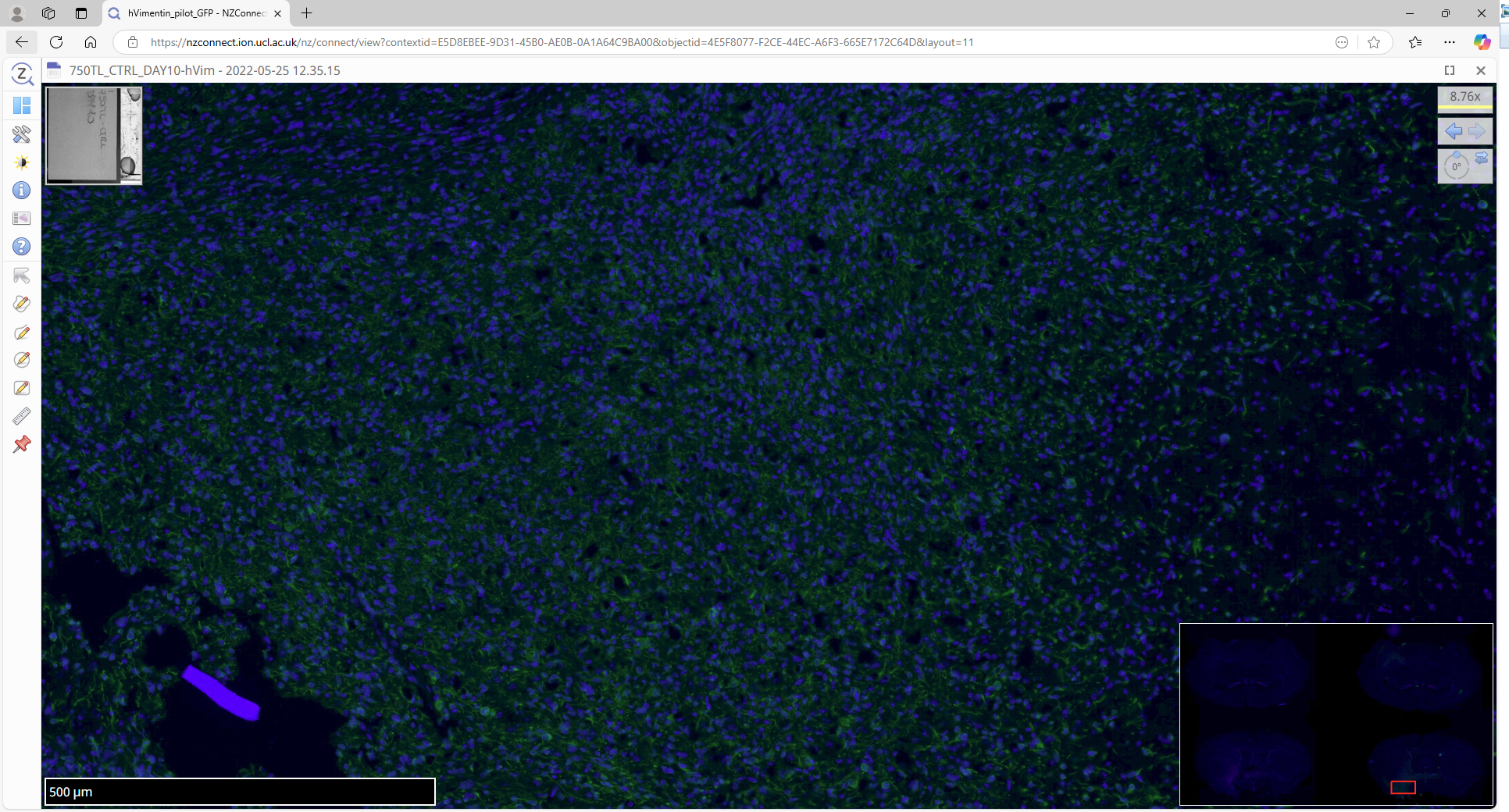

Supplement: Supplementary file 5 — EV Figure Source Data [file 44321_2025_237_MOESM5_ESM.zip › Figure EV2/EV2A/hVim_fromNZC/Day10_CTRL.PNG]

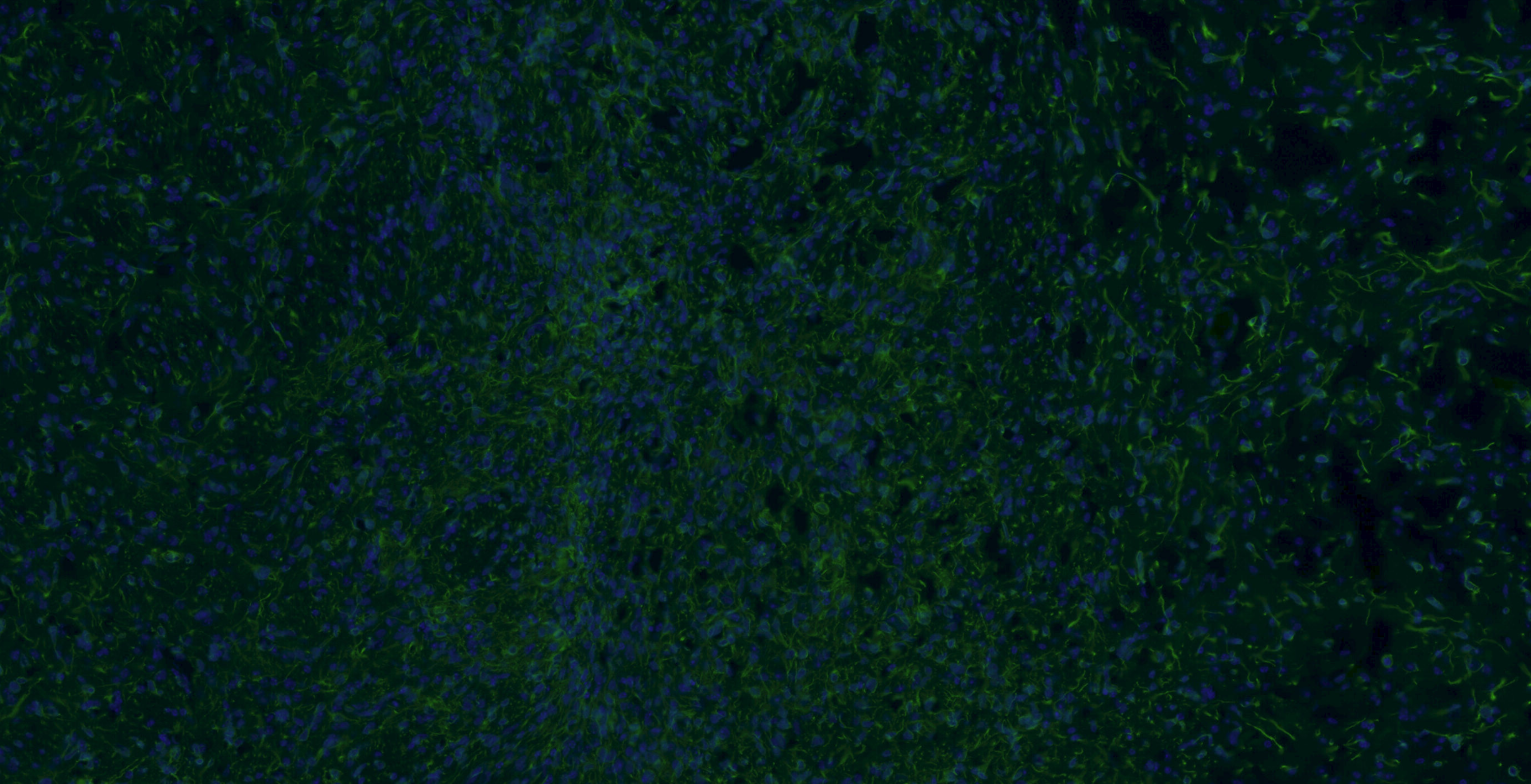

Supplement: Supplementary file 5 — EV Figure Source Data [file 44321_2025_237_MOESM5_ESM.zip › Figure EV2/EV2A/hVim_fromNZC/768BL_TMZ25_DAY5-hVim - 2022-05-25 18.44.55.jpg]

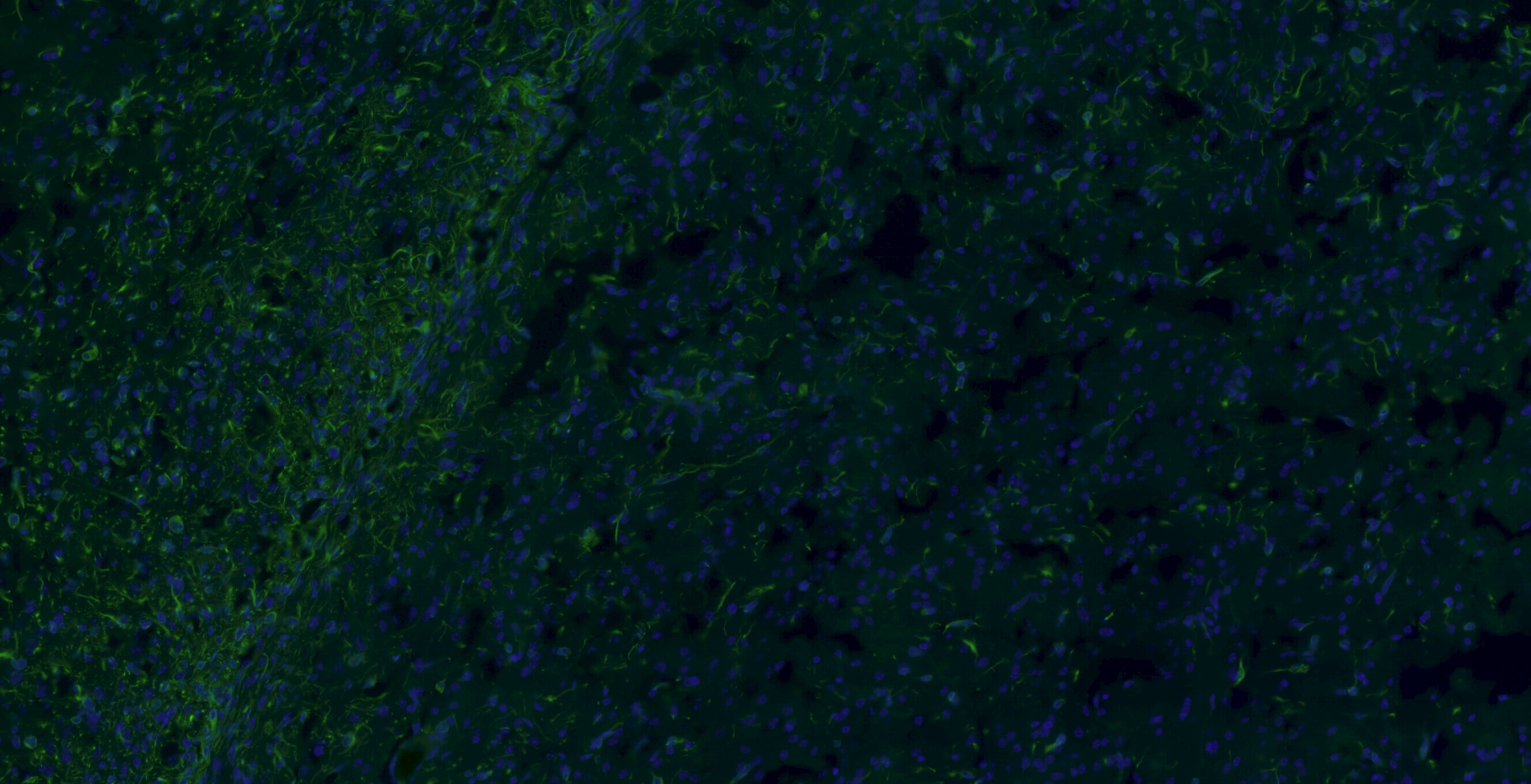

Supplement: Supplementary file 5 — EV Figure Source Data [file 44321_2025_237_MOESM5_ESM.zip › Figure EV2/EV2A/hVim_fromNZC/755tl_tmz++_DAY5-hVim - 2022-05-25 13.00.41.jpg]

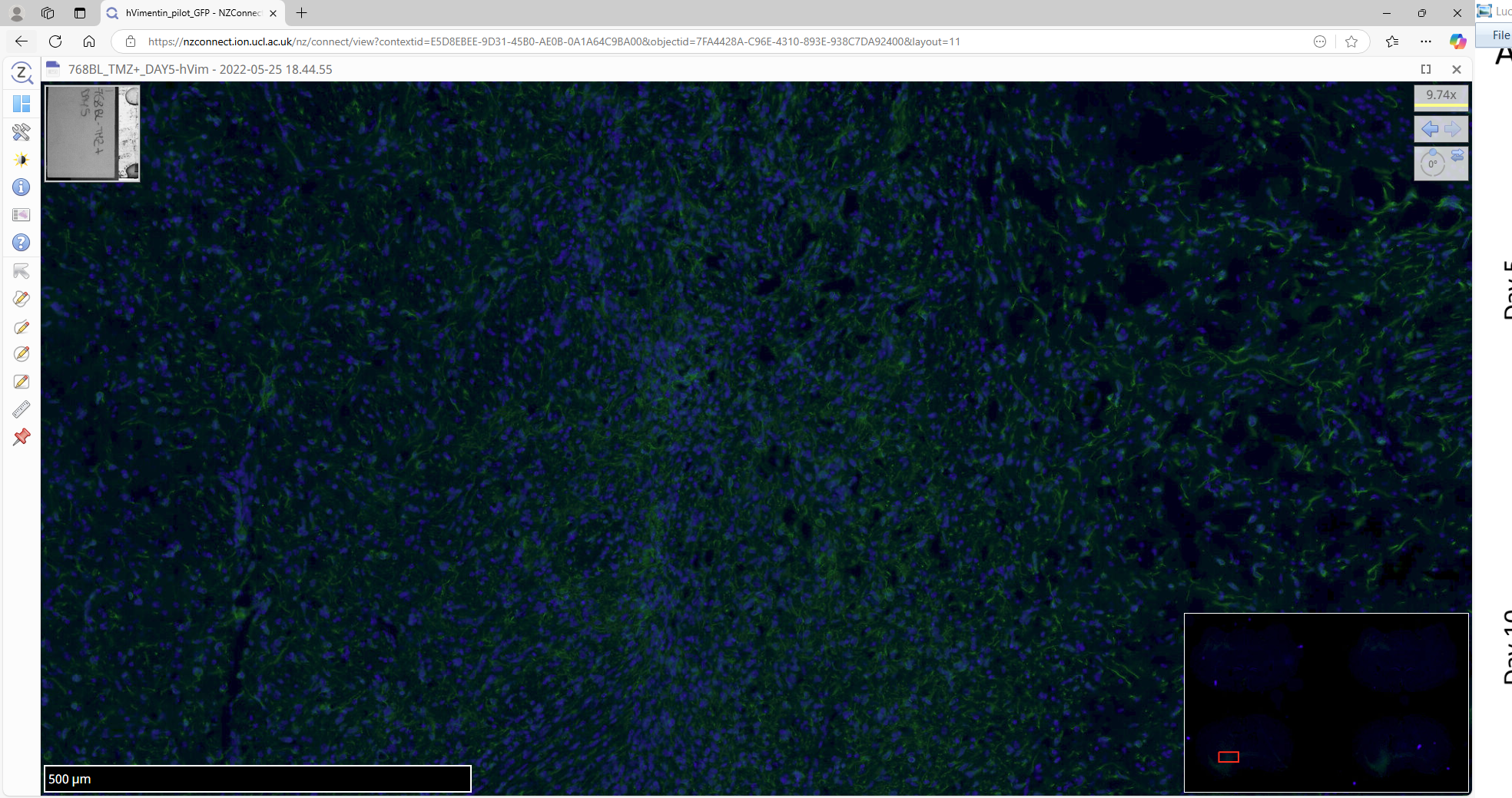

Supplement: Supplementary file 5 — EV Figure Source Data [file 44321_2025_237_MOESM5_ESM.zip › Figure EV2/EV2A/hVim_fromNZC/Day5_TMZ25mg.Kg.PNG]

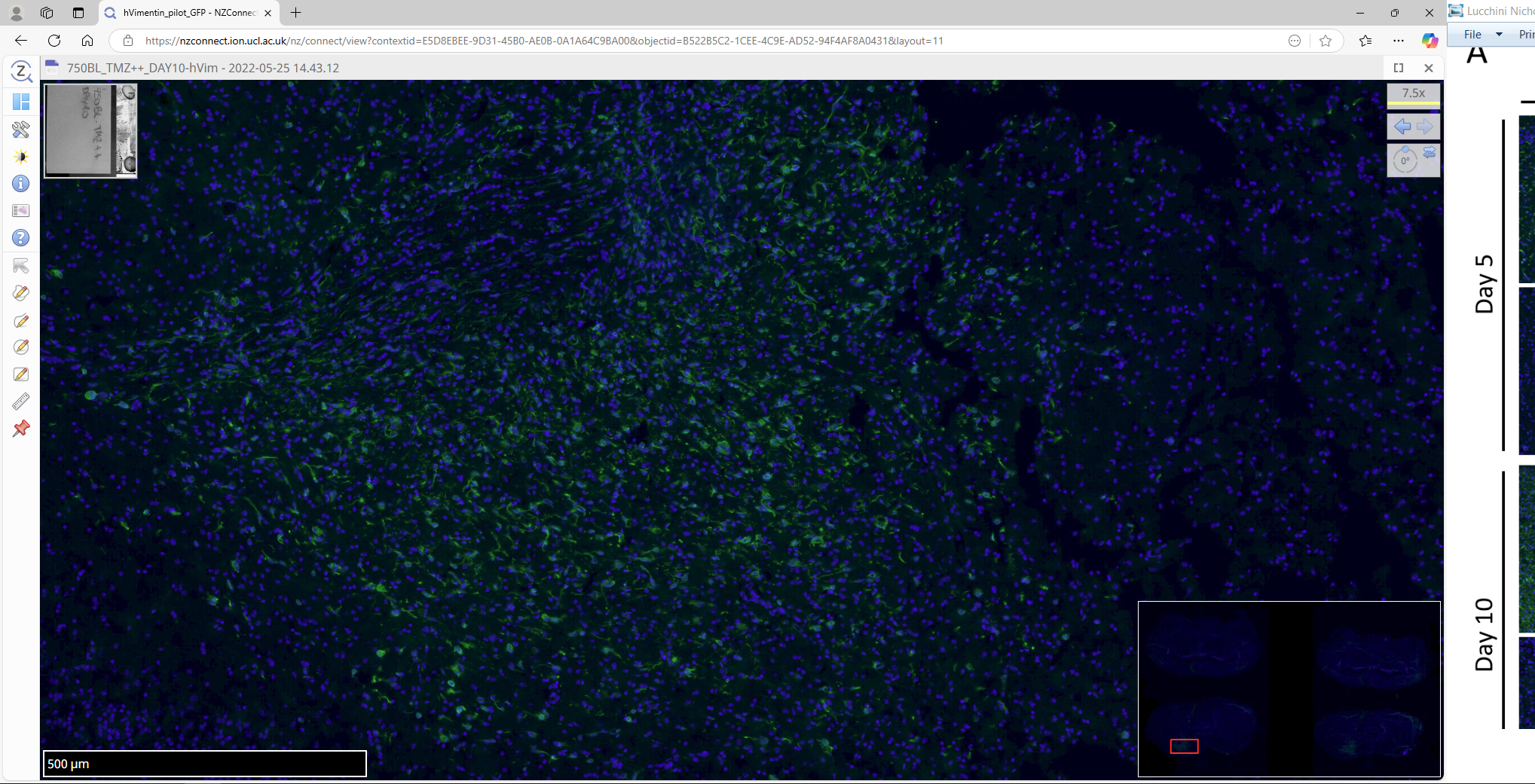

Supplement: Supplementary file 5 — EV Figure Source Data [file 44321_2025_237_MOESM5_ESM.zip › Figure EV2/EV2A/hVim_fromNZC/Day10_TMZ50mg.Kg.PNG]

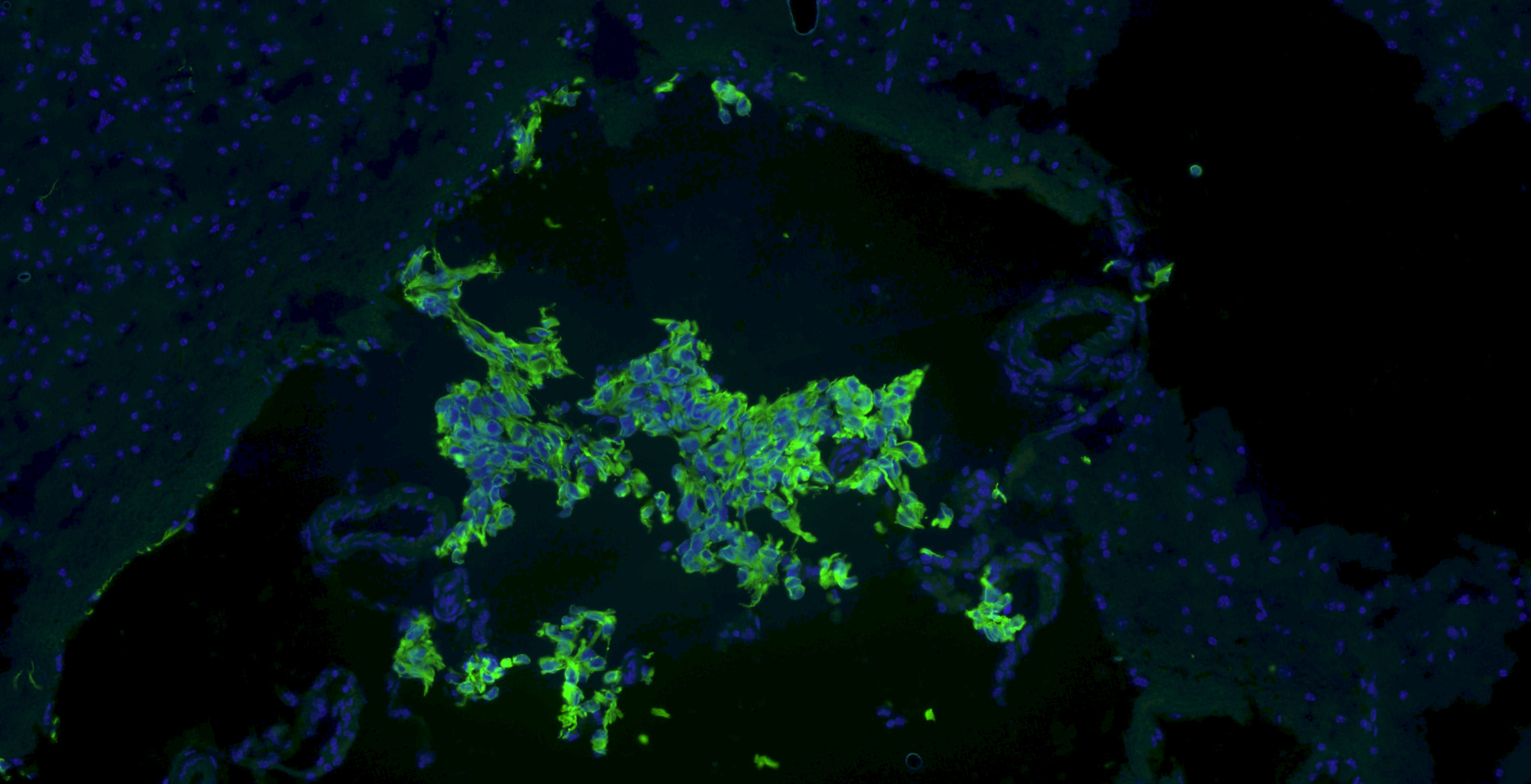

Supplement: Supplementary file 5 — EV Figure Source Data [file 44321_2025_237_MOESM5_ESM.zip › Figure EV2/EV2A/hVim_fromNZC/768 TR_tmz50_DAY5-hVim - 2022-05-25 13.27.03.jpg]

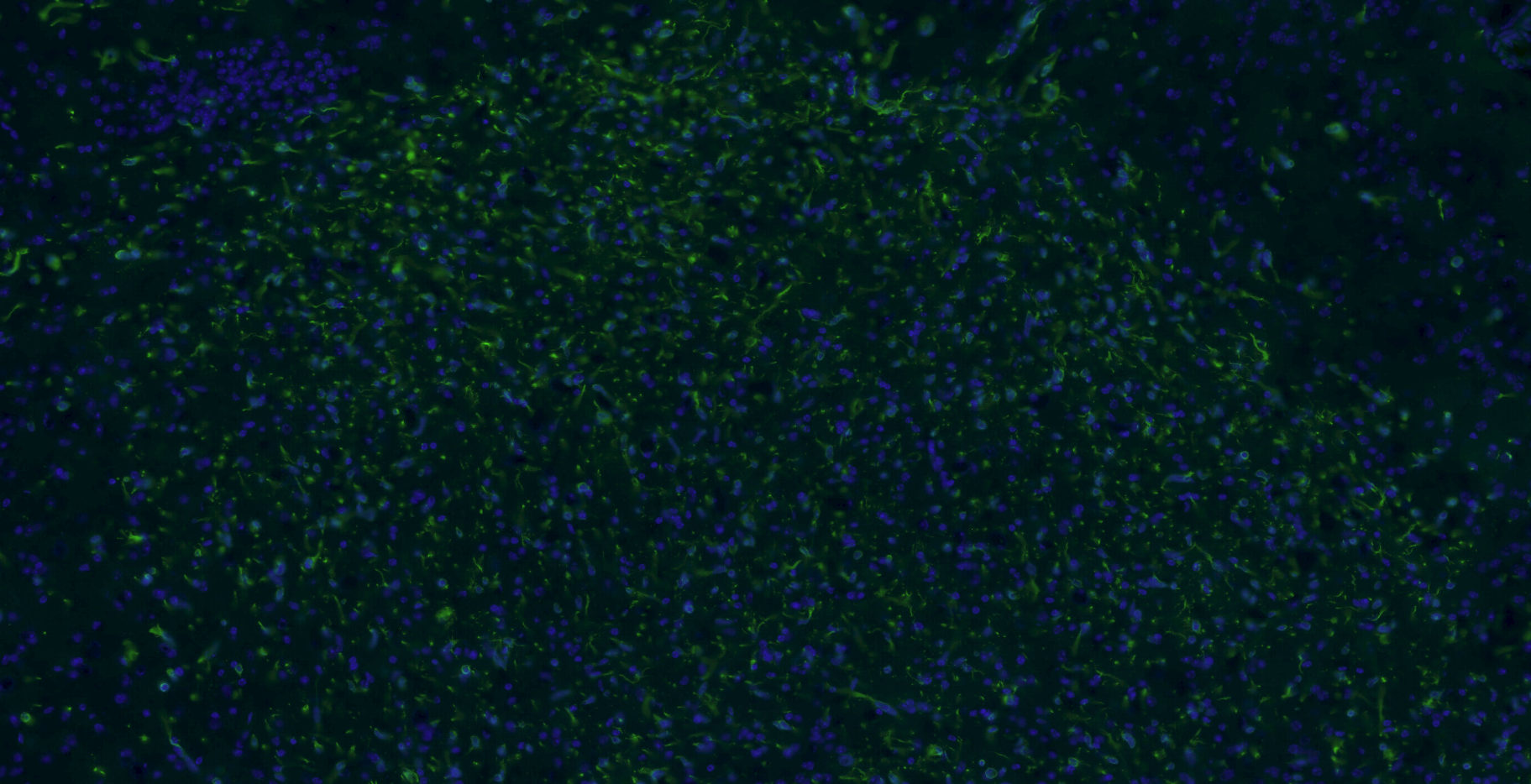

Supplement: Supplementary file 5 — EV Figure Source Data [file 44321_2025_237_MOESM5_ESM.zip › Figure EV2/EV2A/hVim_fromNZC/756TL_CTRL_DAY5-hVim - 2022-05-25 16.23.42.jpg]

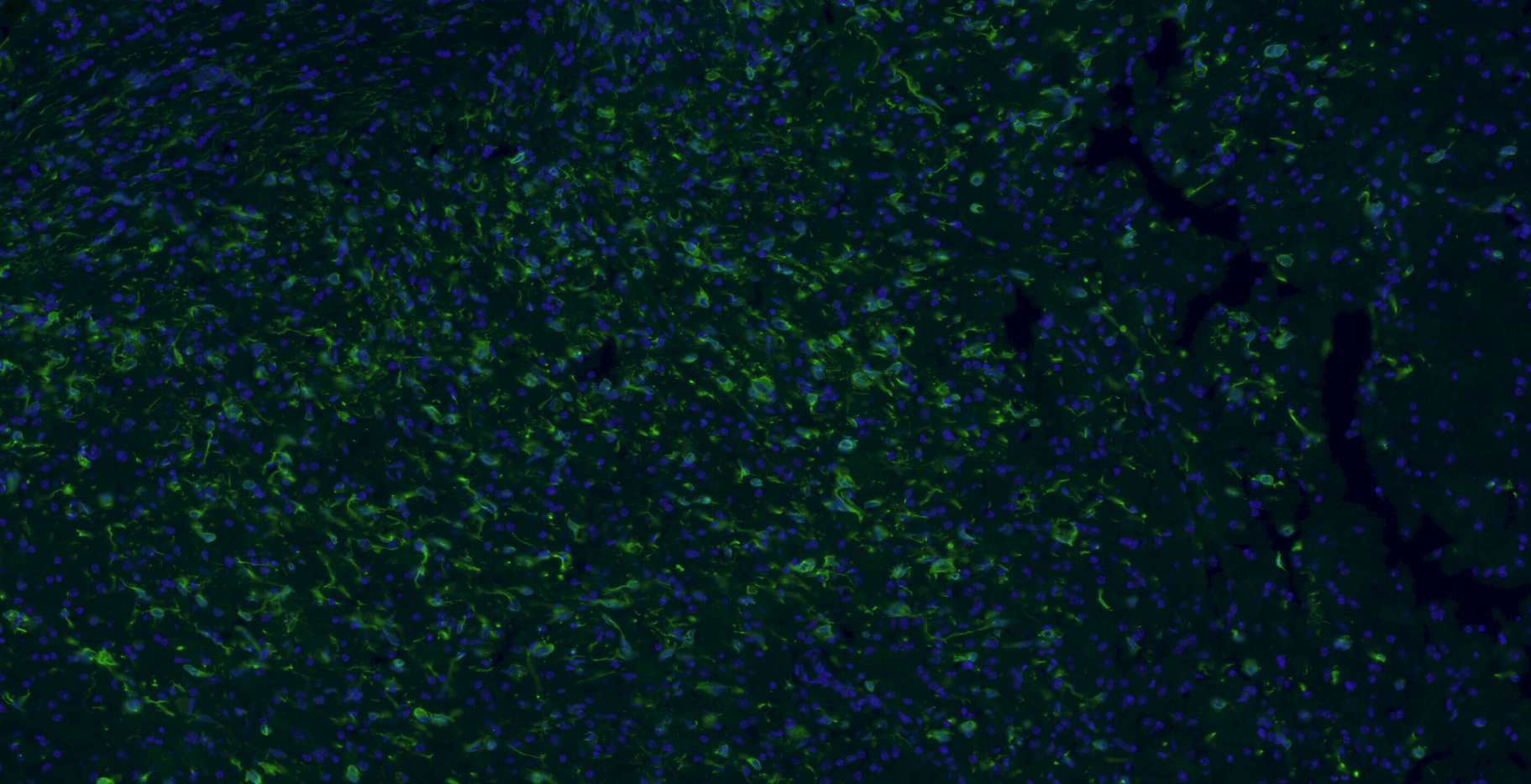

Supplement: Supplementary file 5 — EV Figure Source Data [file 44321_2025_237_MOESM5_ESM.zip › Figure EV2/EV2A/hVim_fromNZC/750BL_TMZ50_DAY10-hVim - 2022-05-25 14.43.12.jpg]

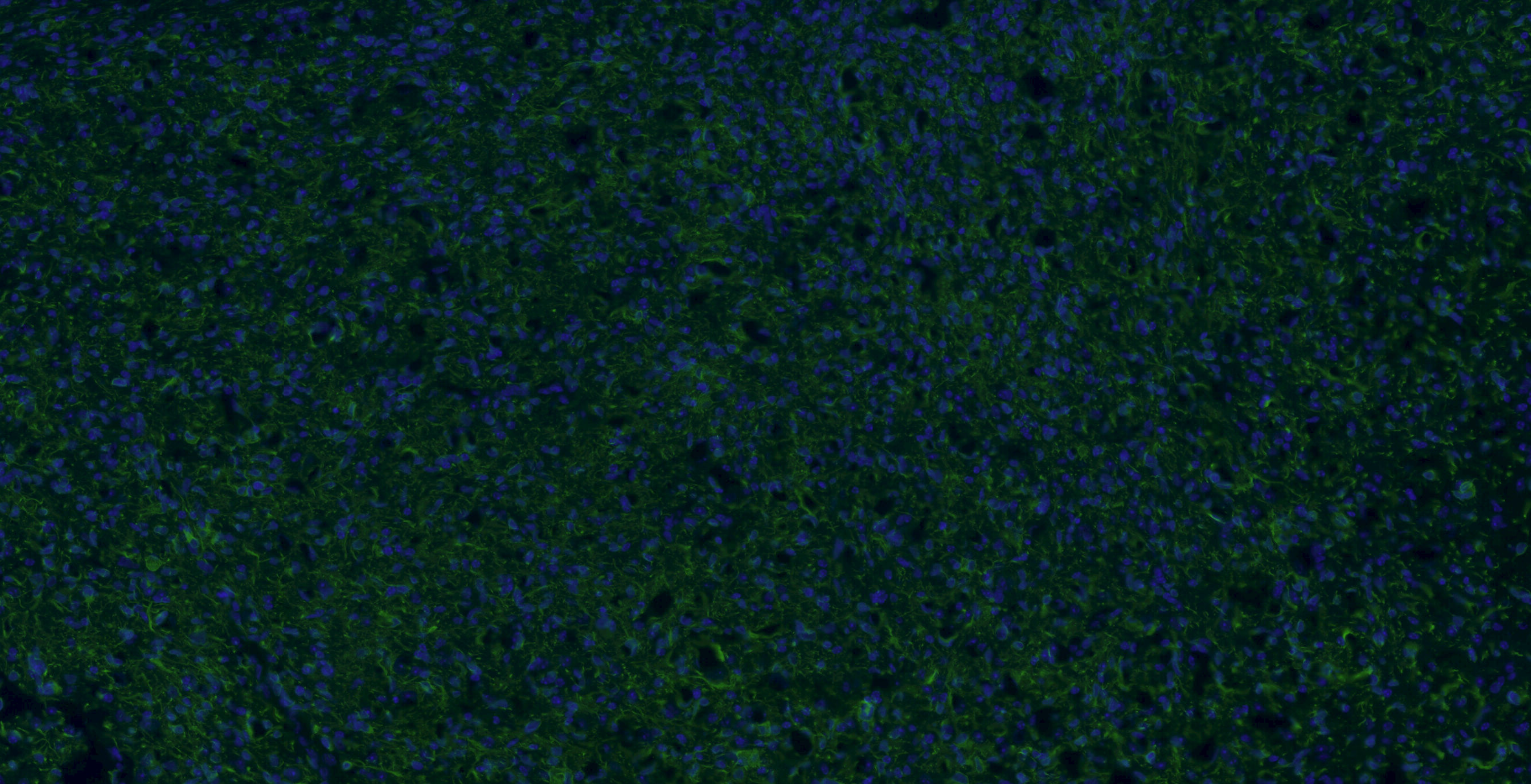

Supplement: Supplementary file 5 — EV Figure Source Data [file 44321_2025_237_MOESM5_ESM.zip › Figure EV2/EV2A/hVim_fromNZC/750TL_CTRL_DAY10-hVim - 2022-05-25 12.35.15.jpg]

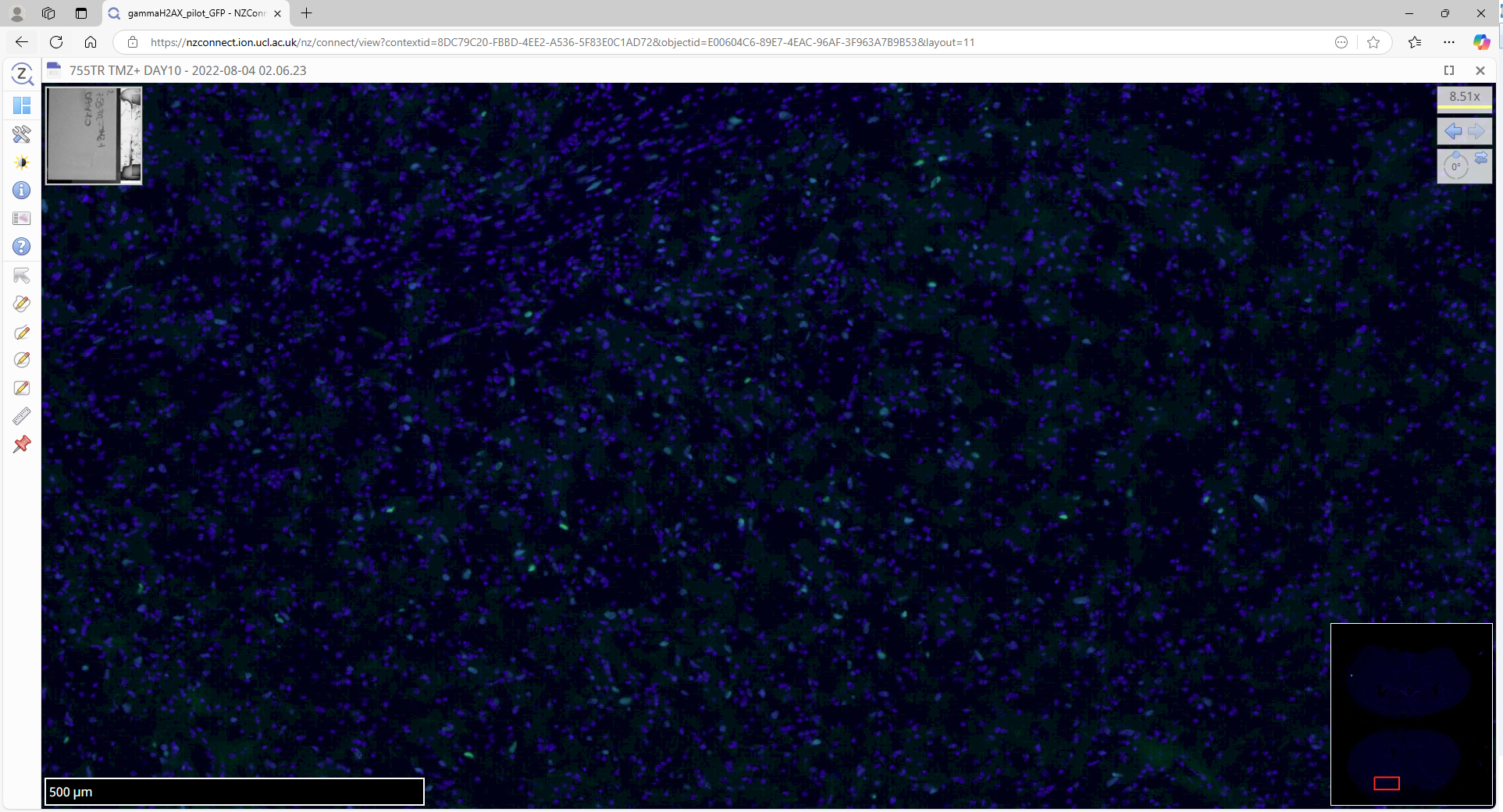

Supplement: Supplementary file 5 — EV Figure Source Data [file 44321_2025_237_MOESM5_ESM.zip › Figure EV2/EV2A/gammaH2AX_fromNZC/Day10_TMZ25mg.Kg.PNG]

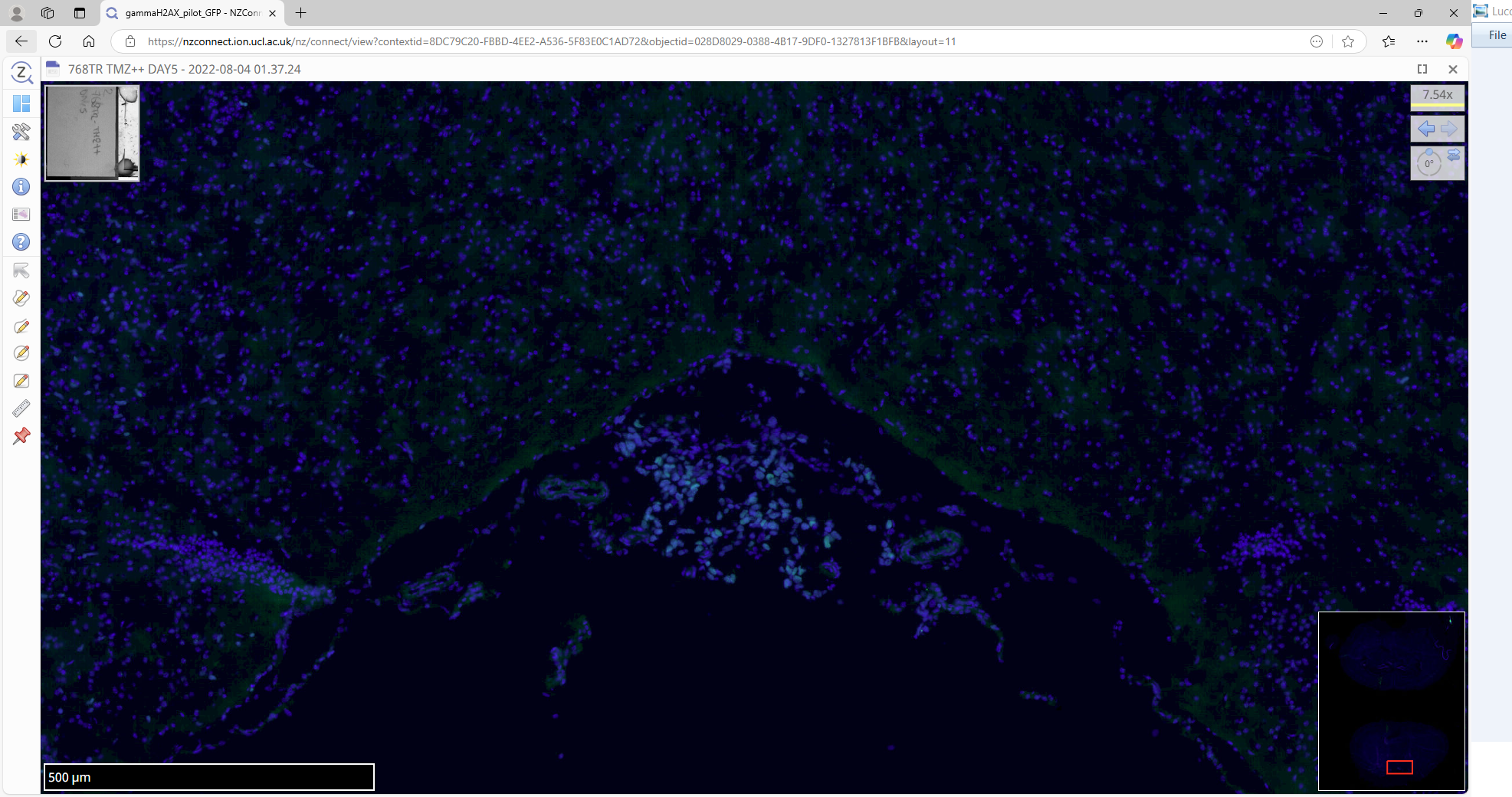

Supplement: Supplementary file 5 — EV Figure Source Data [file 44321_2025_237_MOESM5_ESM.zip › Figure EV2/EV2A/gammaH2AX_fromNZC/Day5_TMZ50mg.Kg.PNG]

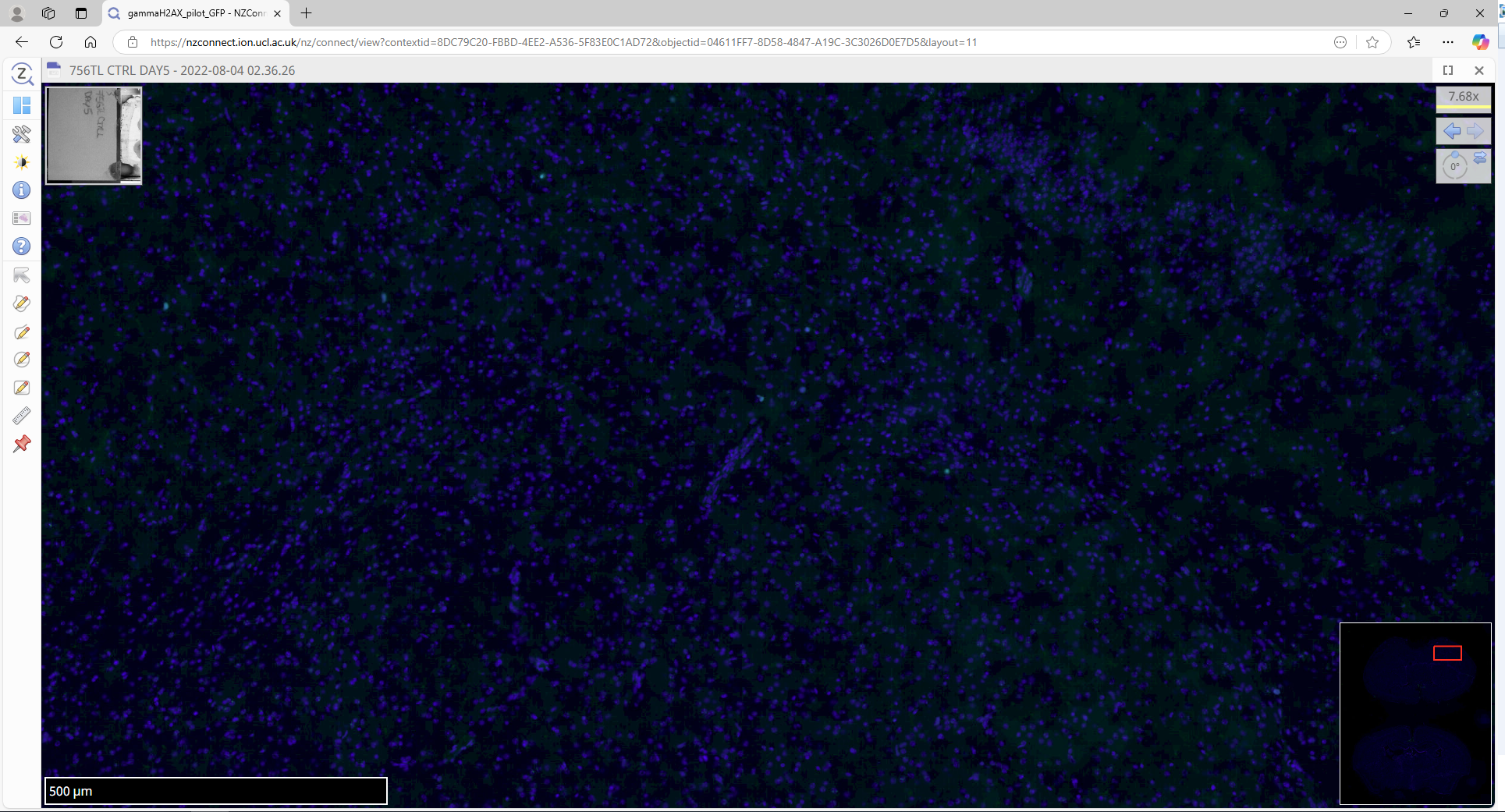

Supplement: Supplementary file 5 — EV Figure Source Data [file 44321_2025_237_MOESM5_ESM.zip › Figure EV2/EV2A/gammaH2AX_fromNZC/Day5_CTRL.PNG]

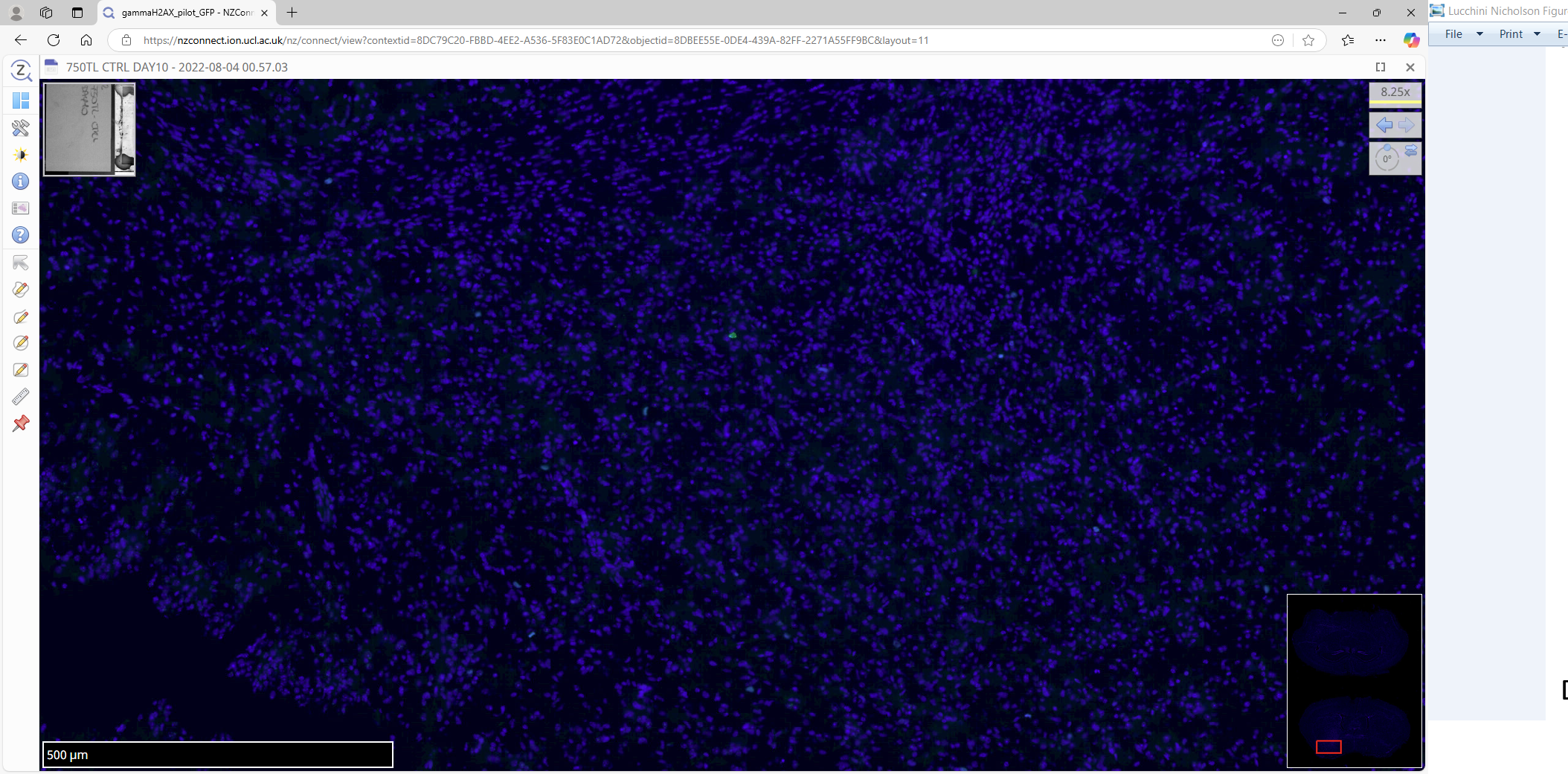

Supplement: Supplementary file 5 — EV Figure Source Data [file 44321_2025_237_MOESM5_ESM.zip › Figure EV2/EV2A/gammaH2AX_fromNZC/Day10_CTRL.PNG]

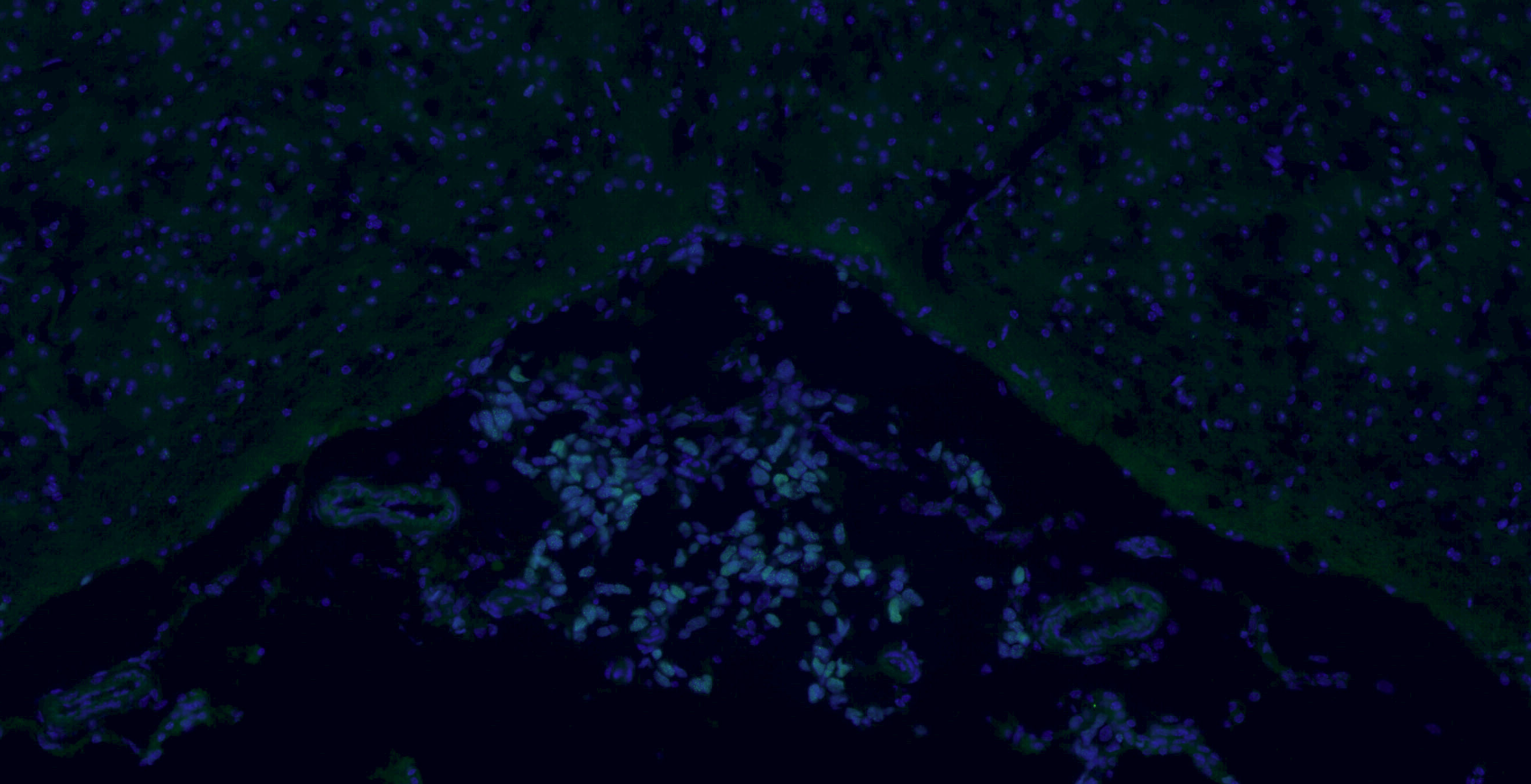

Supplement: Supplementary file 5 — EV Figure Source Data [file 44321_2025_237_MOESM5_ESM.zip › Figure EV2/EV2A/gammaH2AX_fromNZC/768TR TMZ++ DAY5 - 2022-08-04 01.37.24.jpg]

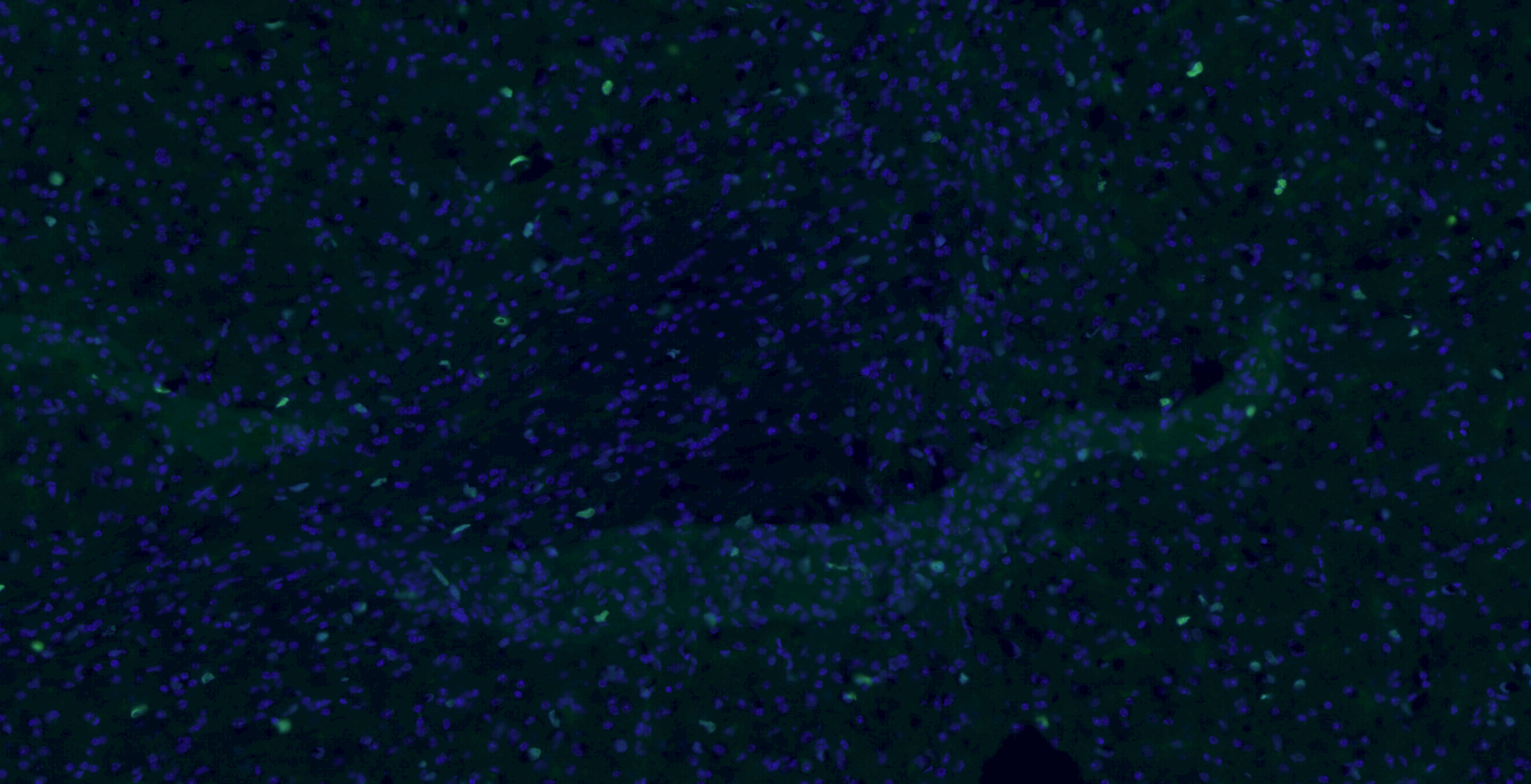

Supplement: Supplementary file 5 — EV Figure Source Data [file 44321_2025_237_MOESM5_ESM.zip › Figure EV2/EV2A/gammaH2AX_fromNZC/750BL TMZ++ DAY10 - 2022-08-04 00.43.54.jpg]

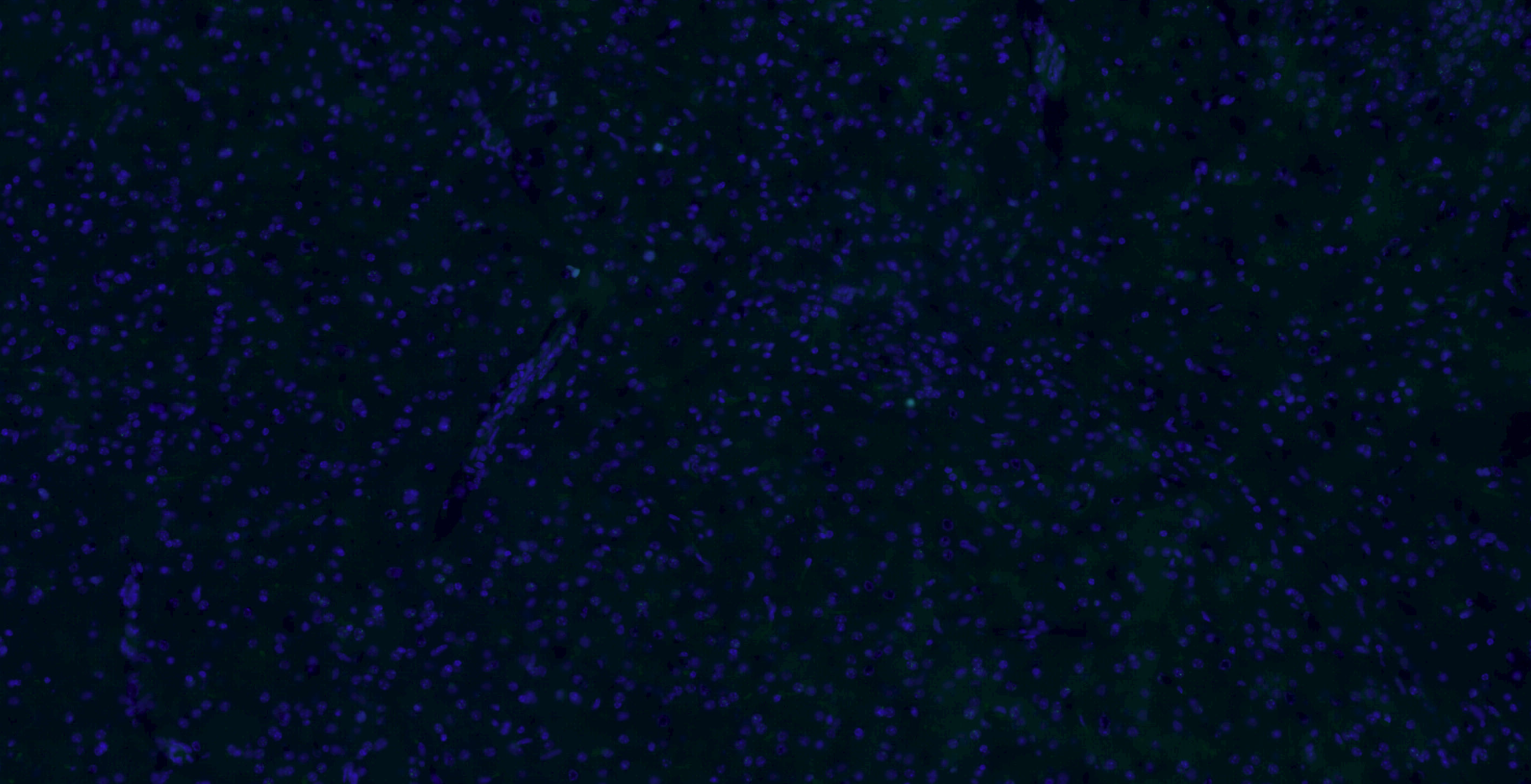

Supplement: Supplementary file 5 — EV Figure Source Data [file 44321_2025_237_MOESM5_ESM.zip › Figure EV2/EV2A/gammaH2AX_fromNZC/756TL CTRL DAY5 - 2022-08-04 02.36.26.jpg]

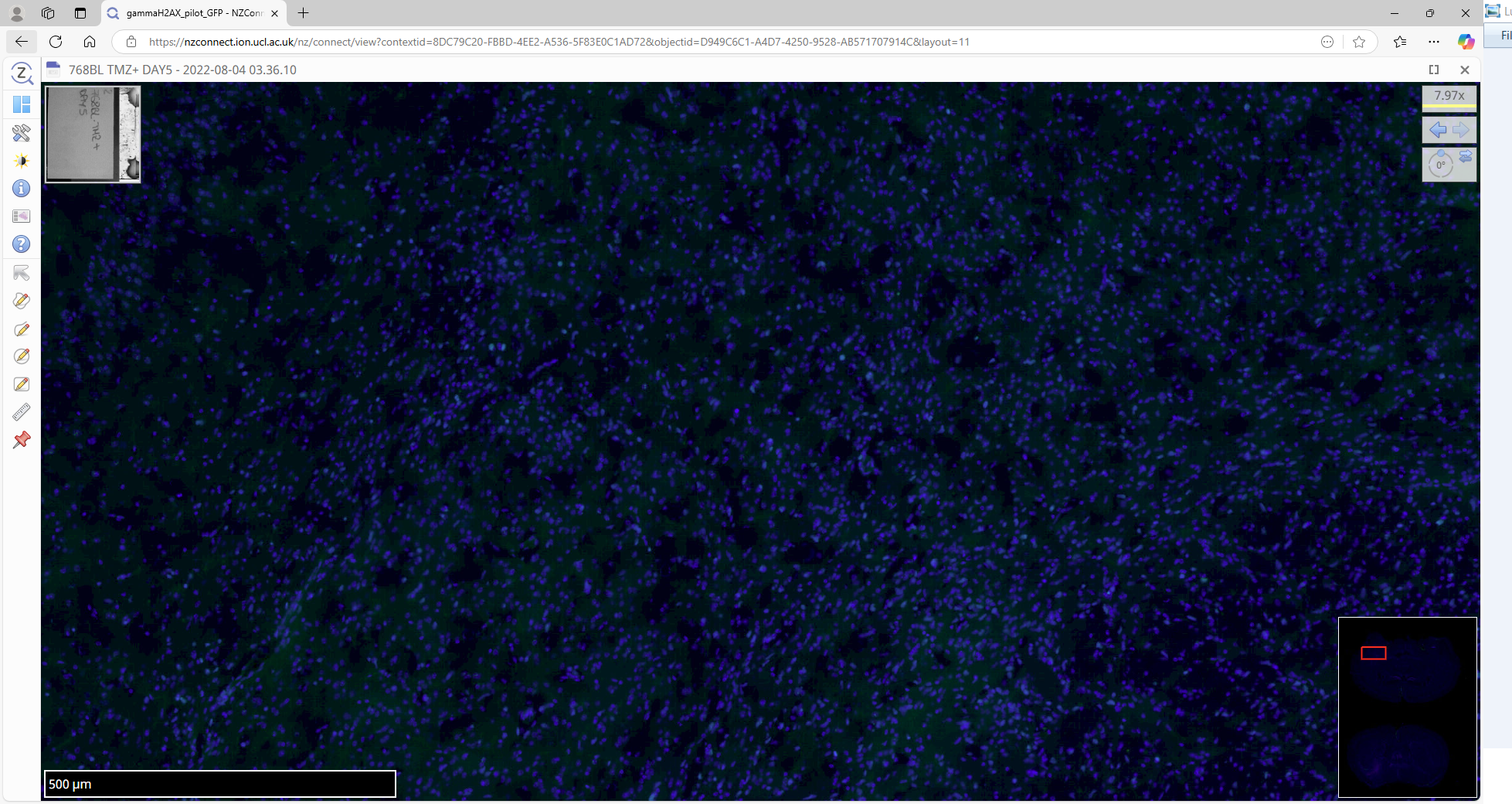

Supplement: Supplementary file 5 — EV Figure Source Data [file 44321_2025_237_MOESM5_ESM.zip › Figure EV2/EV2A/gammaH2AX_fromNZC/Day5_TMZ25mg.Kg.PNG]

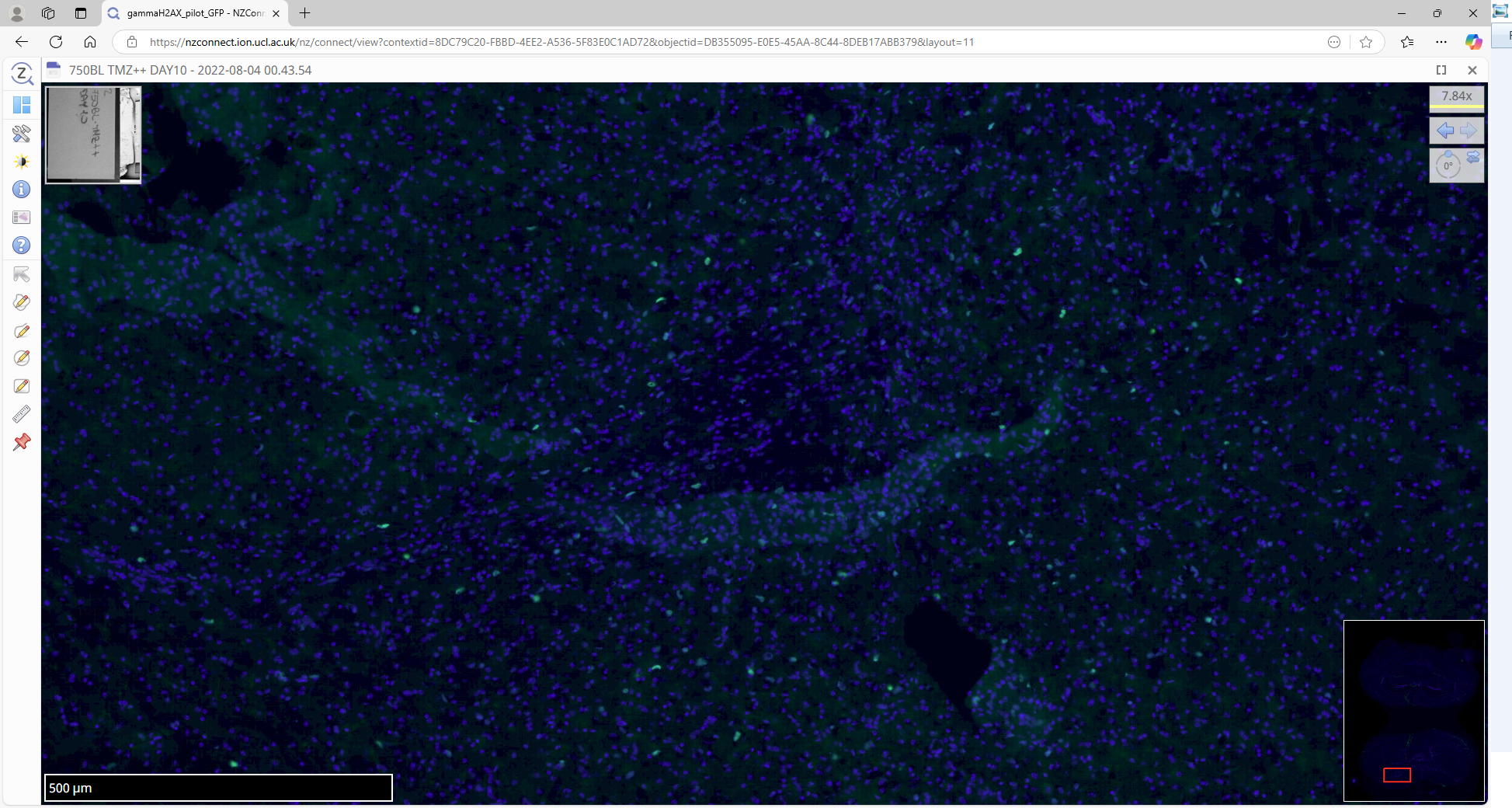

Supplement: Supplementary file 5 — EV Figure Source Data [file 44321_2025_237_MOESM5_ESM.zip › Figure EV2/EV2A/gammaH2AX_fromNZC/Day10_TMZ50mg.Kg.PNG]

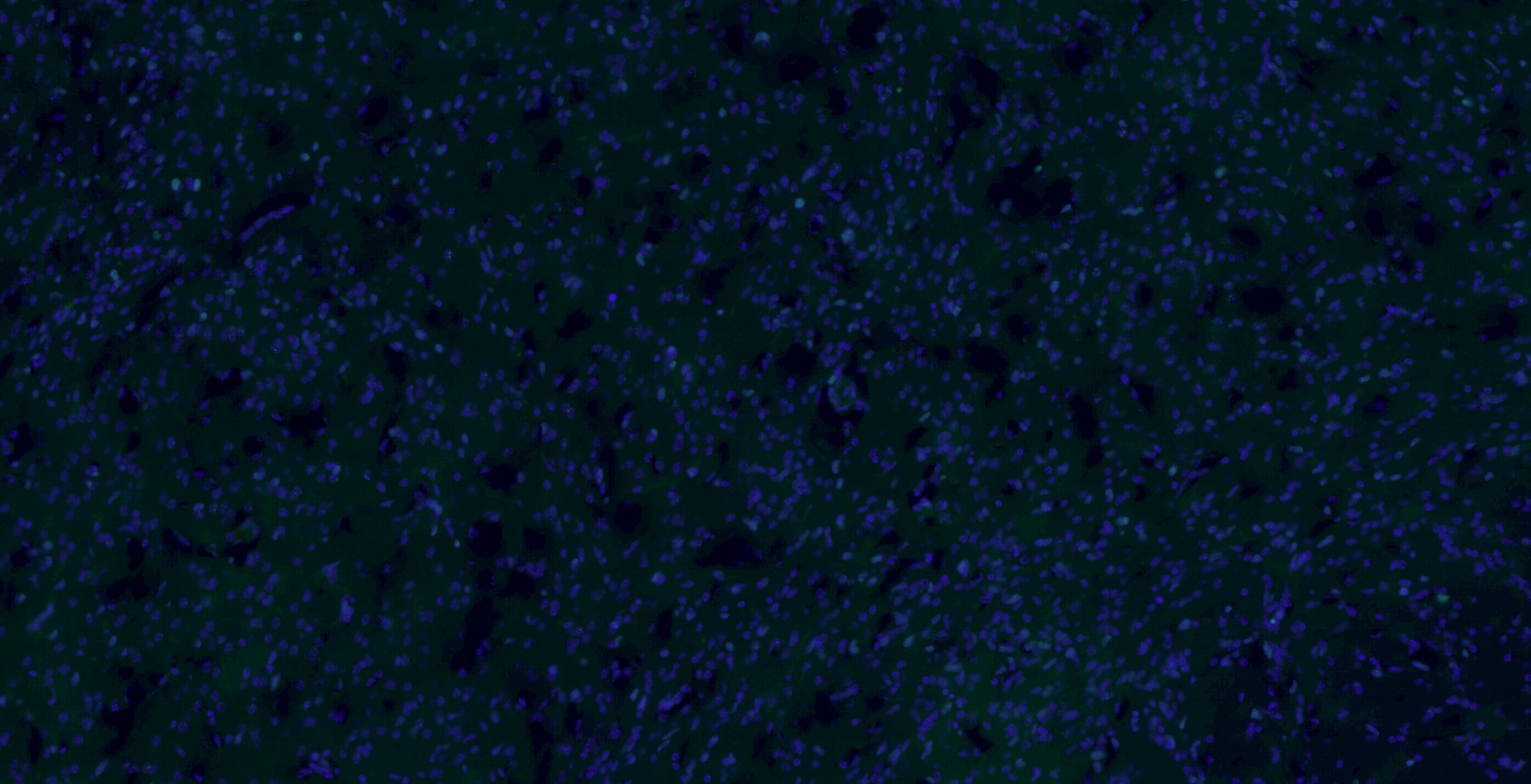

Supplement: Supplementary file 5 — EV Figure Source Data [file 44321_2025_237_MOESM5_ESM.zip › Figure EV2/EV2A/gammaH2AX_fromNZC/768BL TMZ+ DAY5 - 2022-08-04 03.36.10.jpg]

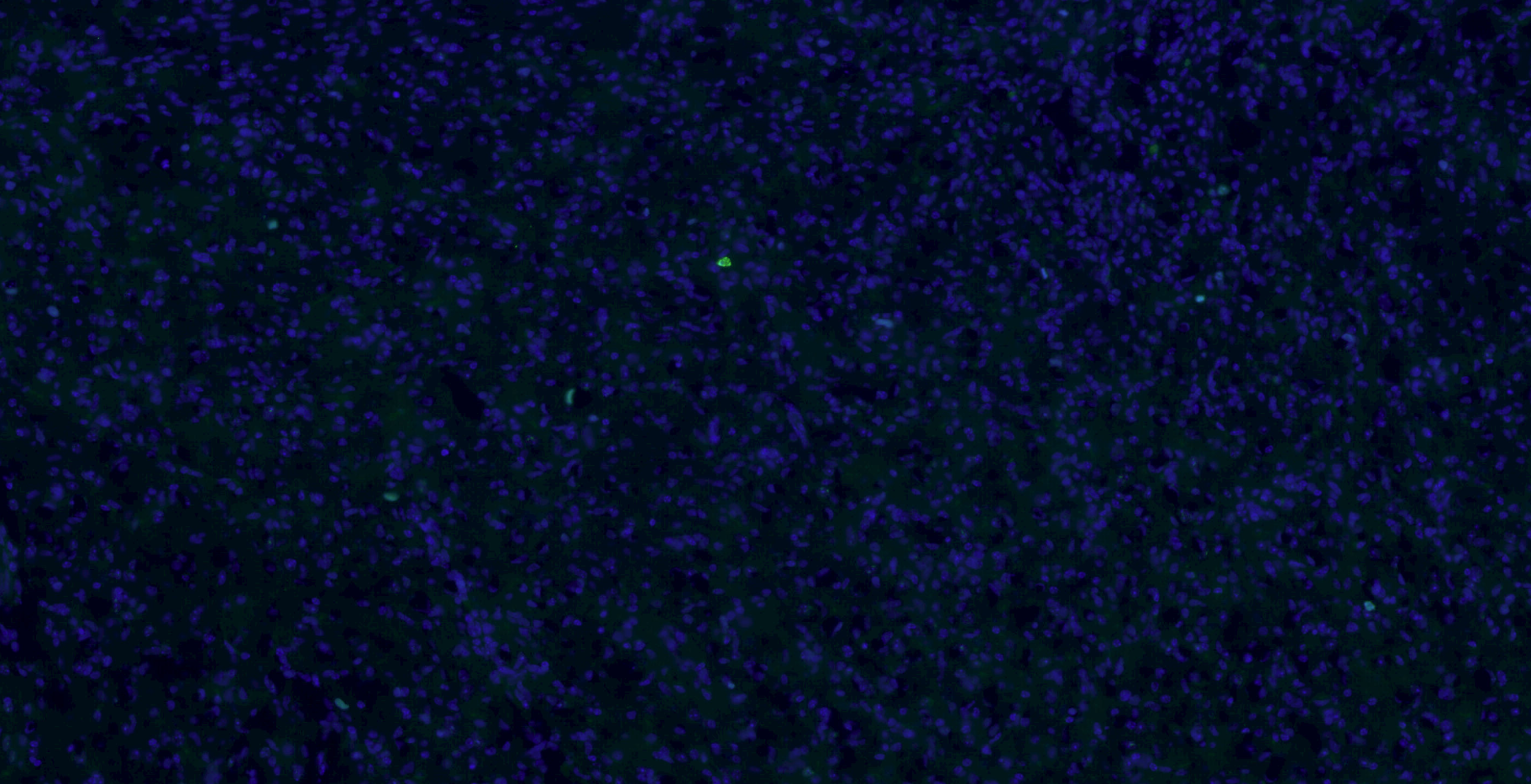

Supplement: Supplementary file 5 — EV Figure Source Data [file 44321_2025_237_MOESM5_ESM.zip › Figure EV2/EV2A/gammaH2AX_fromNZC/750TL CTRL DAY10 - 2022-08-04 00.57.03.jpg]

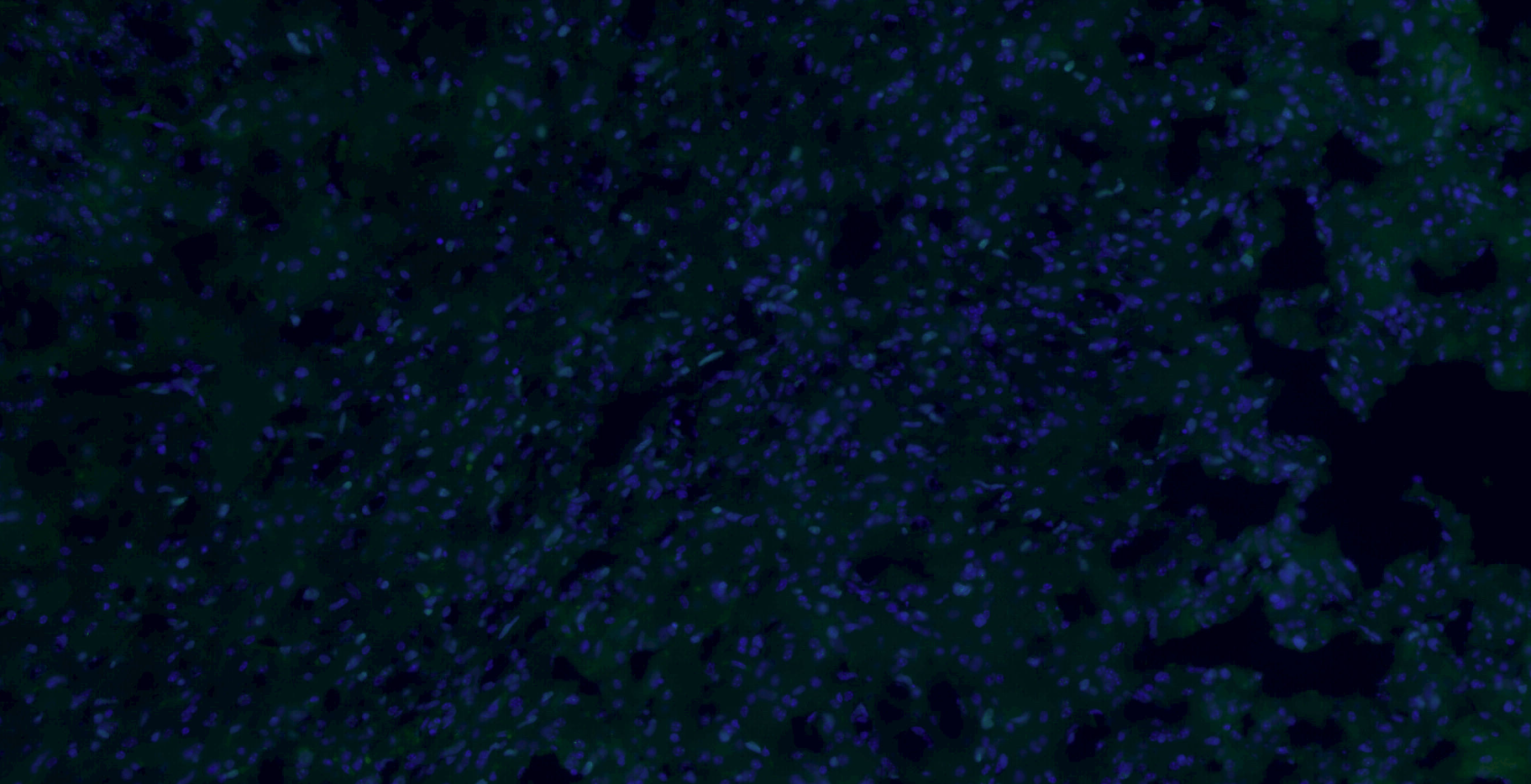

Supplement: Supplementary file 5 — EV Figure Source Data [file 44321_2025_237_MOESM5_ESM.zip › Figure EV2/EV2A/gammaH2AX_fromNZC/755TL TMZ++ DAY5 - 2022-08-04 02.50.25.jpg]

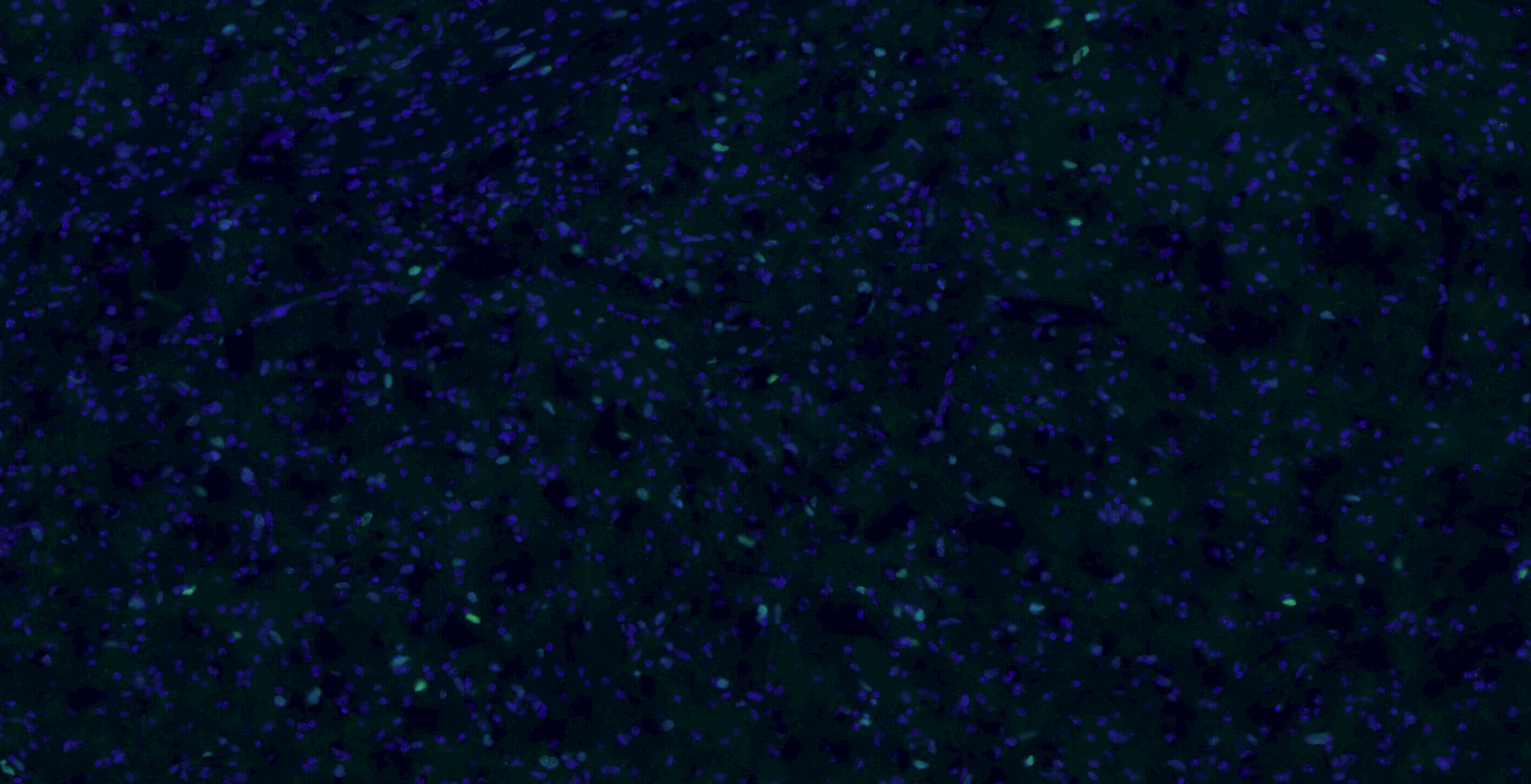

Supplement: Supplementary file 5 — EV Figure Source Data [file 44321_2025_237_MOESM5_ESM.zip › Figure EV2/EV2A/gammaH2AX_fromNZC/755TR TMZ+ DAY10 - 2022-08-04 02.06.23.jpg]
