# Supplementary material for: A novel model of glioblastoma recurrence to identify therapeutic vulnerabilities
Source: EMBO Mol Med. 2025 Apr 28;17(6):1325–54. doi: 10.1038/s44321-025-00237-z (PMC12162887; doi:10.1038/s44321-025-00237-z)
Supplement: Supplementary file 6 — Expanded View Figures [file 44321_2025_237_MOESM6_ESM.pdf]

Expanded View Figures

Figure EV1. Histological characterization of PDX models.

(**A**) Summary table of clinical and histo-molecular features of glioblastoma patients. (**B**) In vitro assessment of successful integration of F-Luciferase reporter genes in each GIC line used for this study. Left: quantification of Luminescence counts after imaging the cells with IVIS and measuring the ROIs ($n = 2$ for all lines; $n = 1$ for negative control). Error bars represent Mean ± SD. Right: 30 s kinetic measurement of firefly luminescence signal with plate reader ($n = 1$, with each line being measured with and without luciferin addition. Neg CTRL is represented by the average of all GIC without luciferin addition). (**C**) Curves showing the average tumour growth, measured via BLI, for all PDX derived from the injection of longitudinal primary and recurrent GICs: on the left both GBM39 and GBM39R ($n = 2$ and $n = 3$, respectively), while on the right GBM67 and GBM67R ($n = 3$). Error bars represent Mean ± SD. Each mouse's signal was normalized to the highest signal measured within the same animal over time. (**D**) Representative ICH staining for hNestin of each untreated PDX model generated in this study. Patient 39 both primary and recurrent tumours at the top and patient 67 at the bottom. Scale bars are 250 µm. (**E**) Representative H&E of both primary untreated PDX models generated in this study highlighting typica malignant features of glioblastoma (XGBM39 at the top and XGBM67 at the bottom). Black arrows indicate vascular proliferation; red arrows indicate mitosis. All images show spread nuclear atypia and high cellularity. Scale bars are 100 µm. (**F**) Representative ICH staining for KI67 (left) and cCaspase3 (right) of each untreated PDX model generated in this study. Patient 39 both primary and recurrent tumours at the top and patient 67 at the bottom. Scale bars are 100 µm.

© The Author(s)

A

Primary-Recurrent Pairs

GBM N	Sex	Age	Location of tumour	Histology Diagnosis	ATRX (IHC)	EGFR	CDKN2A/B	Chr 7	Ch 10	IDH1	IDH2	TERT	MGMT methylation	Methylation class, subclass	Treatment
GBM39	M	71	Right Temporal	Glioblastoma, IDH-wildtype	Retained	No amplification	Deletion	Gain	loss	wt	wt	C228T	unmethylated (MSP PCR)	methylation class glioblastoma, IDH wildtype, subclass RTK I	Aug 2016: RT (60Gy/30#) + TMZ. Feb 2017: adj TMZ. June 2017 2nd surgery.
GBM39R	M	72	Temporal lobe, recurrence	Gliosarcoma, IDH-wildtype	Retained	Not tested	Not tested	Not tested	Not tested	wt	wt	Not tested	unmethylated (MSP PCR)	Not tested	
GBM67	M	51	Right parietal	Glioblastoma, IDH-wildtype	Retained	Amplification	Deletion	Gain	loss	wt	wt	C228T	Unmethylated (Array)	methylation class glioblastoma, IDH wildtype, subclass RTK II	Mar 2017: RT (60Gy/30#) + TMZ. May 2017: adj TMZ. Feb 2018: 2nd surgery.
GBM67R	M	52	Right parietal	Recurent glioblastoma, IDH-wildtype	Not tested	Not tested	Not tested	Not tested	Not tested	wt	wt	Not tested	unmethylated (MSP PCR)	Not tested	

MGMT methylated control

GBM N	Sex	Age	Location of tumour	Histology Diagnosis	ATRX (IHC)	EGFR	CDKN2A/B	Chr 7	Ch 10	IDH1	IDH2	TERT	MGMT methylation	Methylation class, subclass
GIC80	F	23	R Frontal	Glioblastoma, IDH-wildtype	Not tested	Amplification	Deletion	Gain	loss	wt	wt	Not tested	Methylated (array)	methylation class glioblastoma, IDH wildtype, subclass RTK II

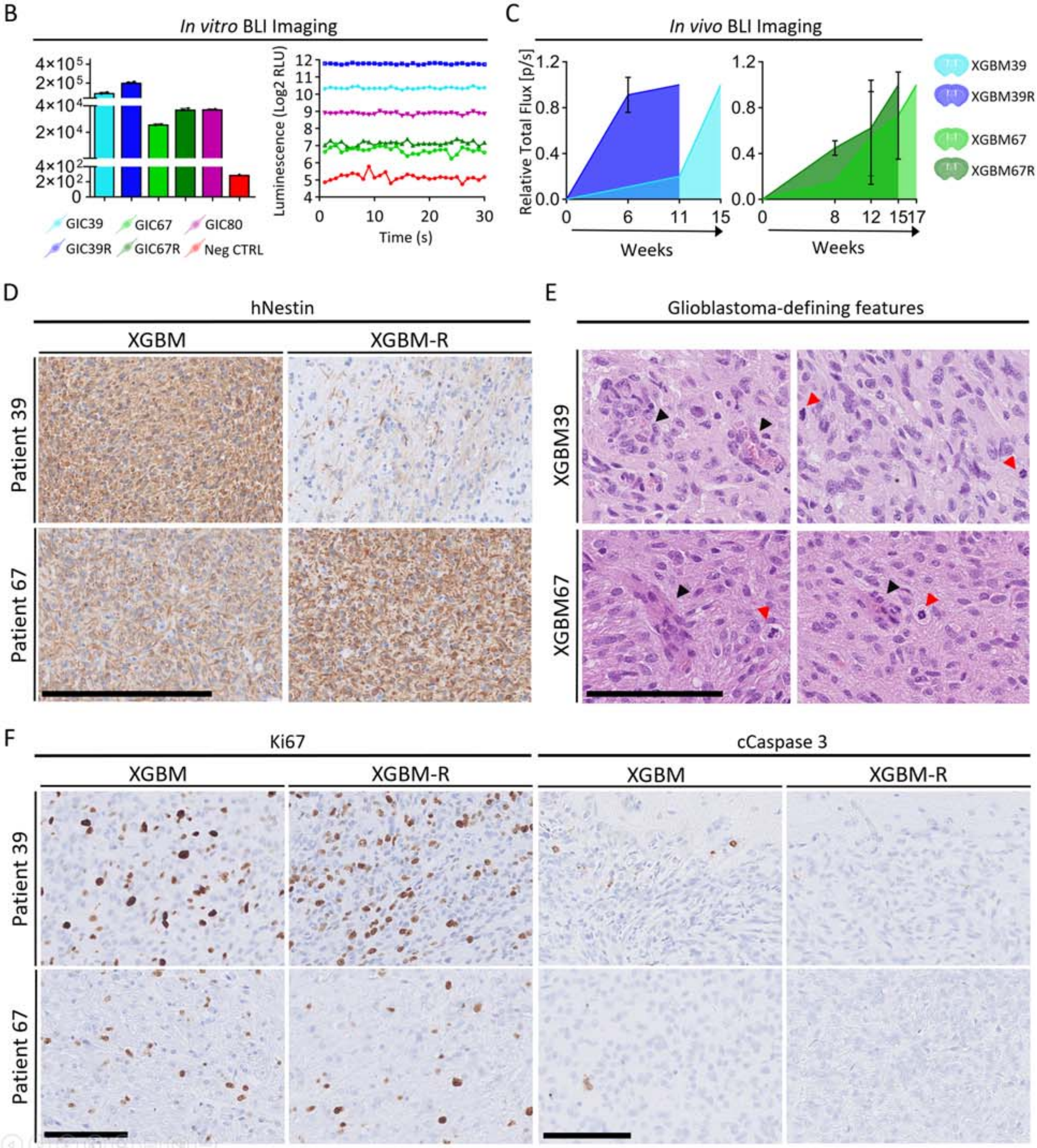

 © The Author(s)

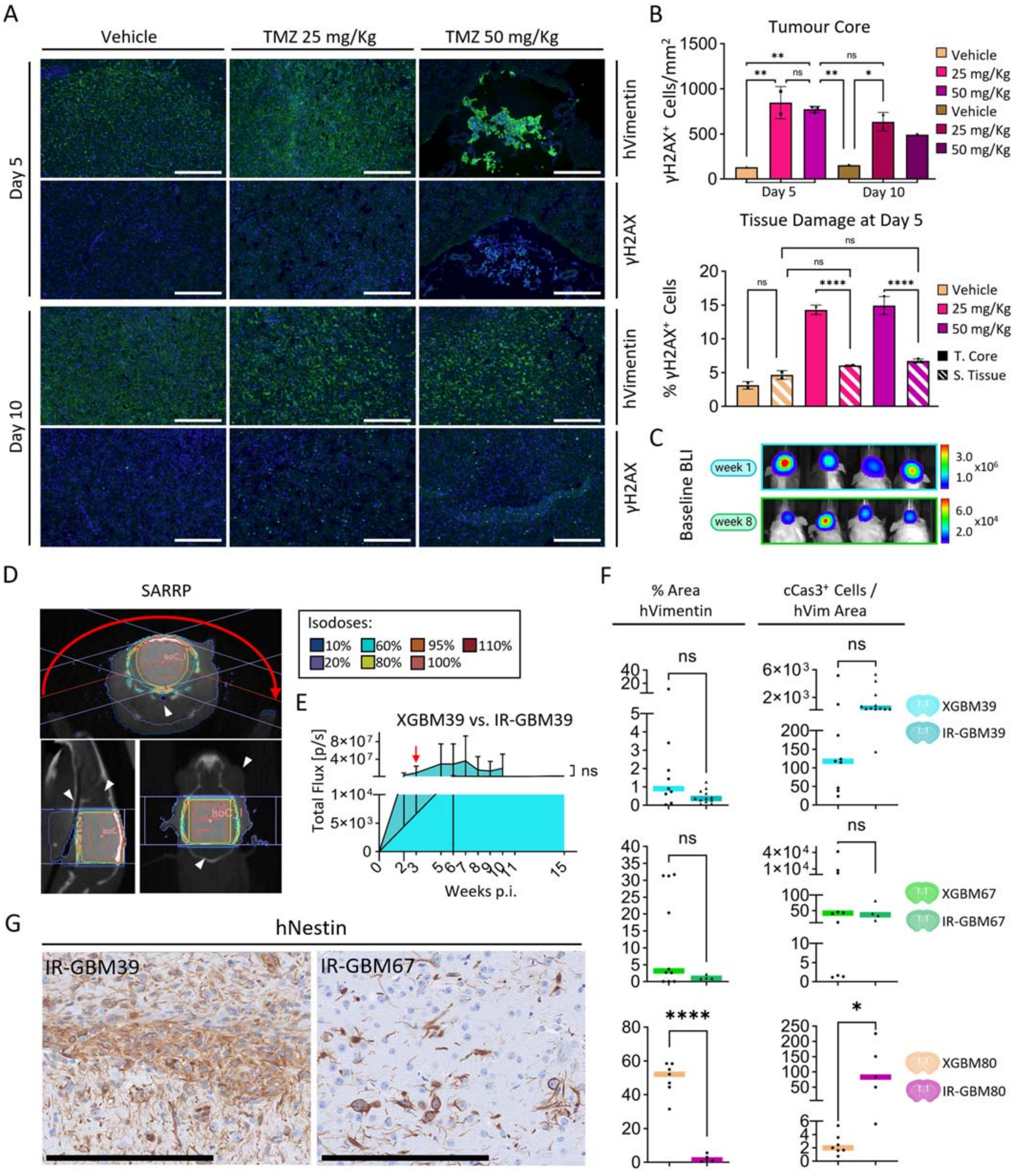
A
Vehicle
TMZ 25 mg/Kg
TMZ 50 mg/Kg
Day 5
Day 10
hVimentin
γH2AX
B
Tumour Core
γH2AX⁺ Cells/mm²
Day 5
Day 10
Vehicle
25 mg/Kg
50 mg/Kg
Tissue Damage at Day 5
% γH2AX⁺ Cells
T. Core
S. Tissue
C
Baseline BLI
week 1
week 8
D
SARRP
Isodoses:
10%
20%
60%
80%
95%
100%
110%
E
XGBM39 vs. IR-GBM39
Total Flux [p/s]
Weeks p.i.
F
% Area hVimentin
cCas3⁺ Cells / hVim Area
XGBM39
IR-GBM39
XGBM67
IR-GBM67
XGBM80
IR-GBM80
G
hNestin
IR-GBM39
IR-GBM67

Figure EV2. Optimization of PDX model of induced recurrence.

(**A**) TMZ tolerability assay: hVimentin and γH2AX IF stainings of brain sections for each time point and each administered dose. Scale bars are 250 μm. (**B**) Quantification of DNA damage at timepoints and doses of TMZ, tumour core and surrounding tissue are shown. Top: DNA damage quantification within the tumour core for each treatment group after 5 and 10 days of continuous administration. $n = 1$ for both vehicles, and Day10 50 mg/kg; $n = 2$ for both Day5 and Day10 25 mg/kg; $n = 3$ for Day5 50 mg/kg. Bottom: comparison of DNA damage between the tumour core and the surrounding tissue as an indication of the toxicity to non-neoplastic cells. $n = 2$ for both vehicles and both 25 mg/kg; $n = 3$ for both 50 mg/kg. One-way ANOVA. *$p = 0.0202$; [25 mg/kg vs. D5 Vehicle]: **$p = 0.0052$; [50 mg/kg vs. D5 Vehicle]: **$p = 0.0062$; [50 mg/kg vs. D10 Vehicle]: **$p = 0.0070$. ****$p < 0.0001$. Error bars represent Mean ± SD. (**C**) IVIS BLI of mice at the chosen timepoint of start of treatment. Each mouse showed a signal at 8 or 1 weeks post injection, respectively, for patients 39 (top) and 67 (bottom). (**D**) Radiation tolerability assay: CT image showing the isodoses of radiation received by the targeted tissue in one representative mouse. White arrows show successful avoidance of radiation-sensitive tissues. (**E**) Comparison of tumour growth curves, measured with BLI, between untreated primary PDXs and treated IR-PDXs derived by the injection of primary GIC39. The red arrow indicates a first scan performed after the sham injection and before radiation and TMZ administration. Error bars represent Mean ± SD. (**F**) Column scatter plots showing the quantification of immunohistochemical staining for hVimentin (%area of brain section surface which is positive for hVim; left) and cCaspase3 (right) in XGBM compared to IR-GBM. $n = 9$ for XGBM39, $n = 12$ for IR-GBM39, $n = 10$ for XGBM67, $n = 4$ for IR-GBM67, $n = 7$ for XGBM80, $n = 5$ for IR-GBM80. Unpaired t-test. ****$p < 0.0001$; *$p = 0.0106$. (**G**) Representative ICH staining for hNestin of both IR-PDX models generated in this study. Patient 39 on the left, patient 67 on the right. Scale bars are 250 μm. Source data are available online for this figure.

 © The Author(s)

A

Sample	Type	Sequencing methodology: WES	DNA-me	RNAseq	scRNAseq
GBM39	FFPE	1	1	1	-
GBM39R	FFPE	1	1	1	-
GIC39	cells	-	2	2	-
GIC39R	cells	-	2	2	-
XGIC39	cells	-	6	6	2
XGIC39R	cells	-	8	8	2
IR_GIC39R	cells	-	-	-	2
XGIC39	FFPE	1	1	1	-
XGIC39R	FFPE	1	1	1	-
GBM67	FFPE	1	1	1	-
GBM6R	FFPE	1	1	1	-
GIC67	cells	-	2	2	-
GIC67R	cells	-	2	2	-
XGIC67	cells	-	8	8	2
XGIC67R	cells	-	8	8	2
IR_GIC67	cells	-	4	4	2
XGIC67	FFPE	1	-	-	-
XGIC67R	FFPE	1	-	-	-
IR_GIC67	FFPE	1	-	-	-

Legend: Patient tumors; Patient-derived GICs; XGICs from PDX models

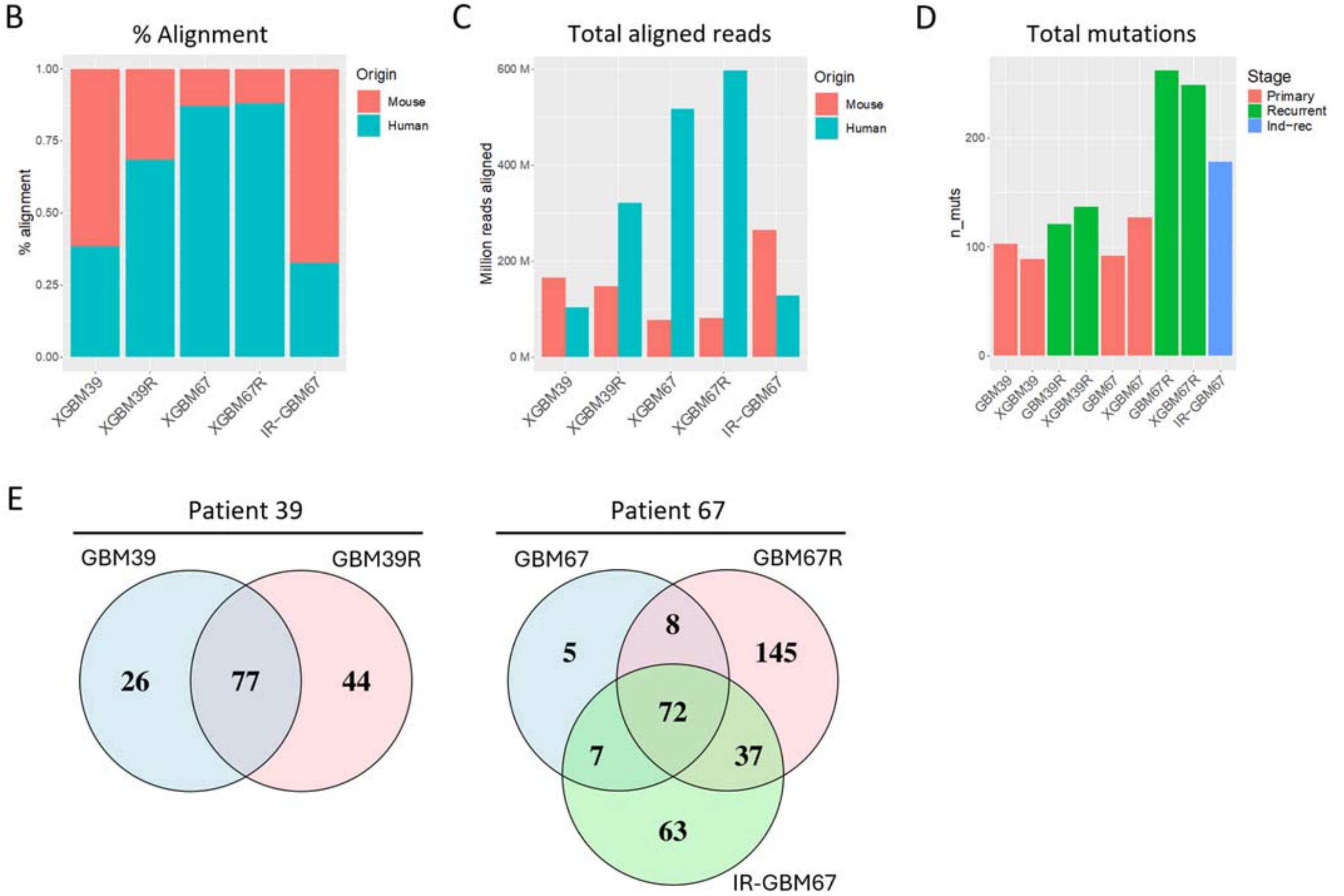

Figure EV3. Whole exome sequencing of patient tumours and PDX models.

(**A**) Summary of GBM samples used for multi-omic characterization of PDX models. (**B**) % of WES reads aligning to human vs mouse genome. (**C**) Total number of reads aligning to human vs mouse proportion of concatenated genome. (**D**) Total number mutations detected in WES samples. (**E**) Venn diagrams showing the number over overlapping mutations between primary, recurrent and modelled recurrence samples in patient 39 (left) and patient 67 (right).

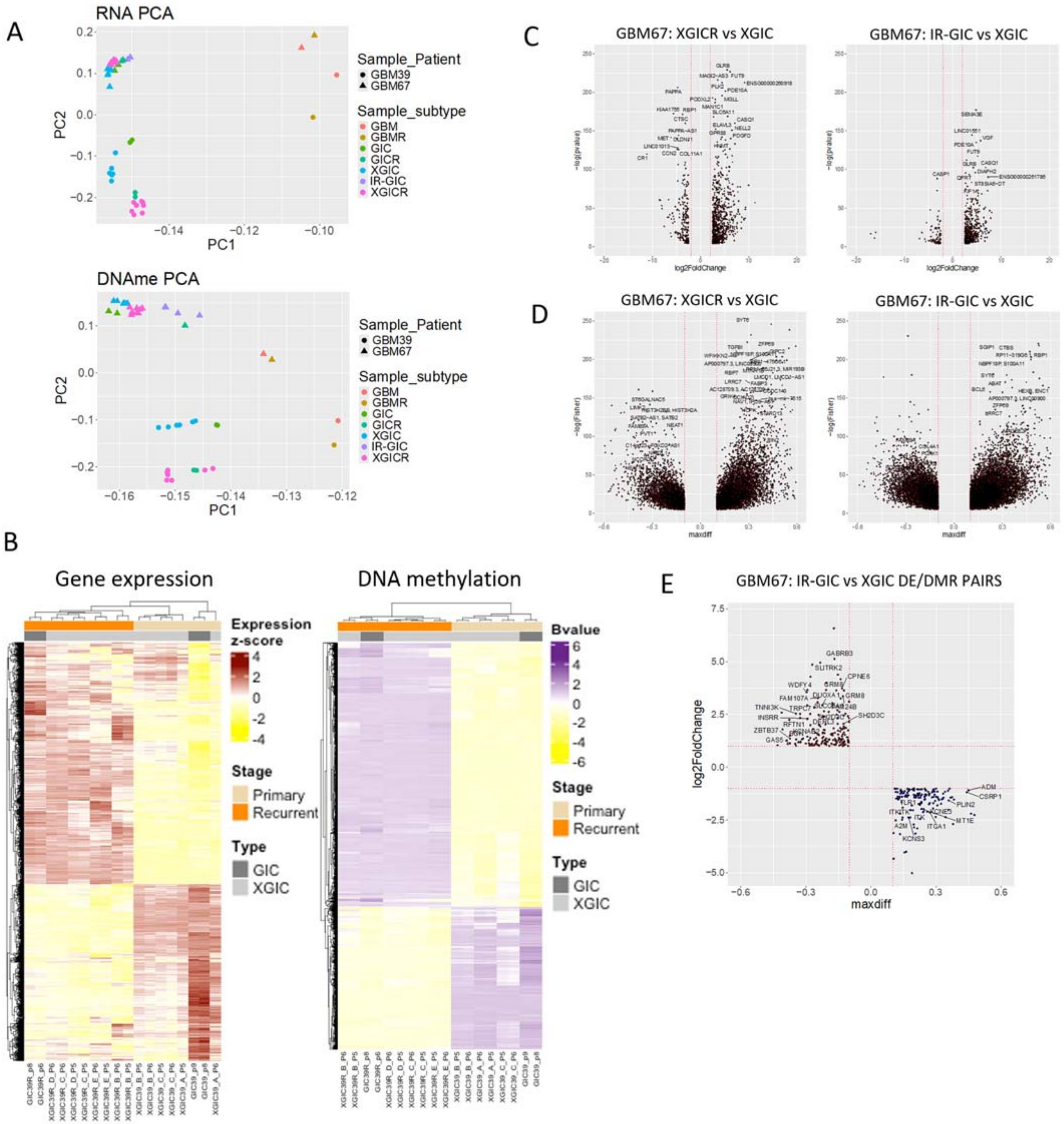

Figure EV4. Transcriptional and epigenetic analysis of PDX models.

(**A**) Principal component analysis of all RNA (top) and DNAme (bottom) GBM and patient- and xenograft-derived GIC samples. Samples are coloured according to their subtype (Primary GBM, recurrent GBM, patient-derived primary GICs or recurrent GICRs, xenograft-derived primary XGIC, induced-recurrence IR-GIC or true recurrence XGICR) and shaped according to their patient of origin with patient 39 shown as circles and patient 67 shown as triangles. (**B**) Semi-supervised hierarchical clustering of RNA (left) and DNAme (right) patient- and xenograft-derived GIC samples from patient 39. (**C**) Volcano plot of differential gene expression between XGIC67 and XGIC67R (left) or between XGIC67 and IR-GIC67 (right) samples. Y-axis shows −log(*P*-value) calculated in DEseq2 using a Wald's test. (**D**) Volcano plot of differential DNA methylation between XGIC67 and XGIC67R (left) or between XGIC67 and IR-GIC67 (right) samples. Y-axis shows -log(Fisher) calculated in DMRcate using a Fishers exact test. (**E**) Scatter plot of the concordantly regulated genes identified between GIC67 vs IR-GIC67 samples. Hypomethylated and overexpressed genes are red, hypermethylated and downregulated genes are blue. Labelled genes were also found in the XGIC vs XGICR comparison.

 © The Author(s)

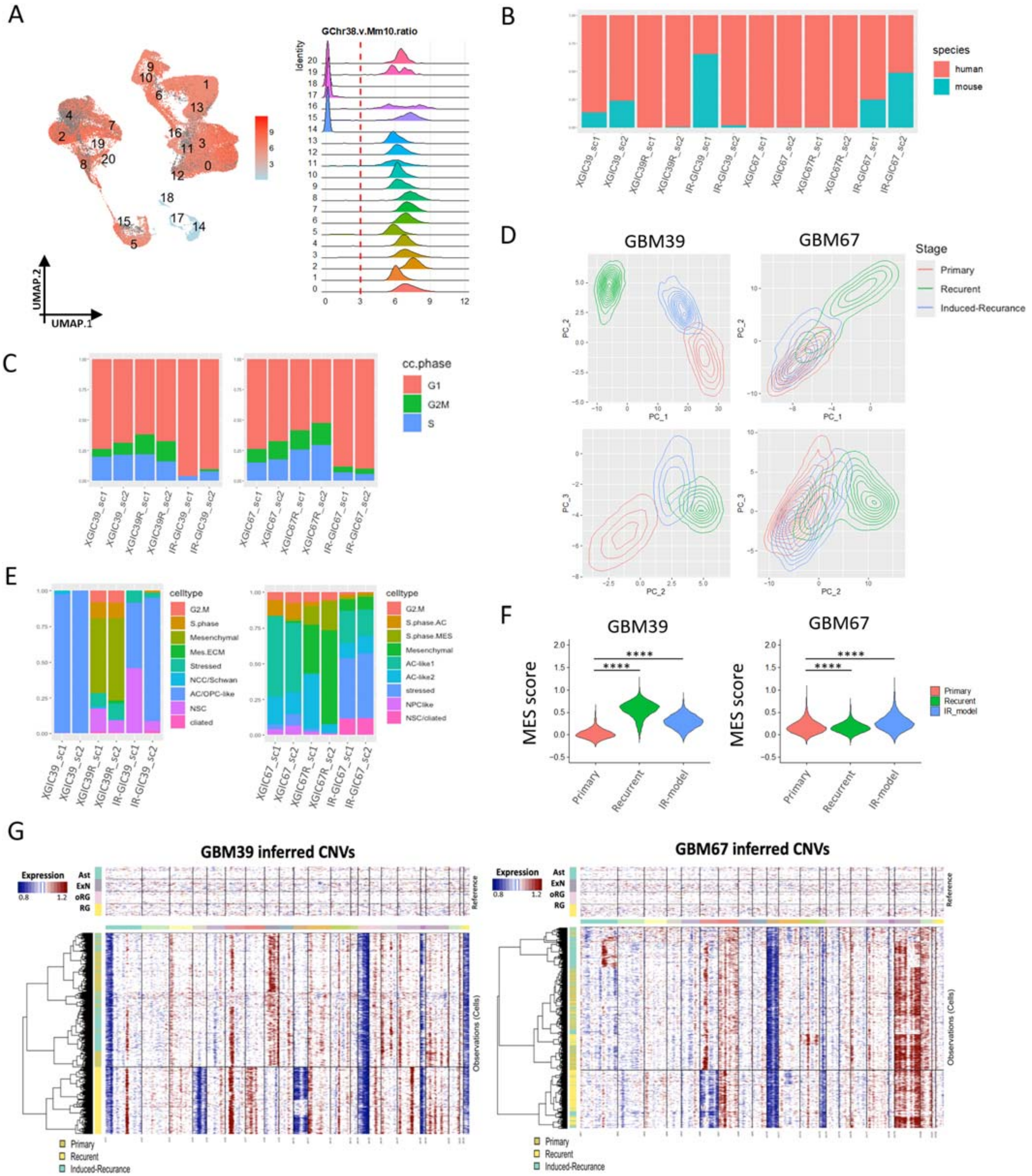
A
GChr38.v.Mm10.ratio
Identity
UMAP.2
UMAP.1
B
species
human
mouse
XGIC39_sc1
XGIC39_sc2
XGIC39R_sc1
XGIC39R_sc2
IR-GIC39_sc1
IR-GIC39_sc2
XGIC67_sc1
XGIC67_sc2
XGIC67R_sc1
XGIC67R_sc2
IR-GIC67_sc1
IR-GIC67_sc2
C
cc.phase
G1
G2M
S
D
GBM39
GBM67
Stage
Primary
Recurent
Induced-Recurance
PC_1
PC_2
PC_3
E
celltype
G2.M
S.phase
Mesenchymal
Mes.ECM
Stressed
NCC/Schwan
AC/OPC-like
NSC
ciliated
S.phase.AC
S.phase.MES
AC-like1
AC-like2
stressed
NPClike
NSC/ciliated
F
GBM39
GBM67
MES score
Primary
Recurent
IR_model
Recurrent
IR-model
G
GBM39 inferred CNVs
GBM67 inferred CNVs
Expression
Ast
ExN
oRG
RG
Reference
Observations (Cells)
Primary
Recurent
Induced-Recurance

Figure EV5. scRNAseq of PDX models.

(**A**) UMAP of PDX samples with cells coloured by ratio of reads aligning to human (GChr39) vs Mouse (Mm10) reference genome (left), ridge plot showing GChr39vsMm10 ratio cutoff used to remove clusters of Mouse cells (right). (**B**). Bar plot showing the proportion of human and mouse cells in each sample. (**C**) Bar plots showing the cell cycle phase proportion of cells in each sample of GBM39 (left) and GBM67 (right). (**D**) PCA plots of PDX cells. (**E**) Bar plots showing the cell type proportion in each sample of GBM39 (left) and GBM67 (right). (**F**) Violin plot showing Neftel et al MES-like signature scores in PDX samples. Wilcoxon test with Bonferroni correction (for GBM9 $n =$ Primary, 3727; Recurrent, 20638 IR-model, 979. For GBM67 $n =$ Primary, 20984; Recurrent 19099, IR-model, 2197). $^{****}p < 0.0001$. (**G**) Heatmap showing inferred Copy Number Variants in down samples PDX cells.

 © The Author(s)
